# Chemical Synthesis of the Sec-To-Cys Homologue of Human Selenoprotein F and Elucidation of Its Disulfide-pairing Mode

**DOI:** 10.3389/fchem.2021.735149

**Published:** 2021-07-29

**Authors:** Peisi Liao, Chunmao He

**Affiliations:** School of Chemistry and Chemical Engineering, South China University of Technology, Guangzhou, China

**Keywords:** selenoprotein, selenoprotein F, chemical protein synthesis, disulfide bond, thioredoxin-like domain

## Abstract

Herein, we document a highly optimized synthesis of the Sec-to-Cys homologue of the human selenoprotein F (SelF) through a three-segment two-ligation semisynthesis strategy. Highlighted in this synthetic route are two one-pot manipulations, i.e. the first ligation followed by a desulfurization and the second ligation followed by the protein refolding. This way multi-milligrams of the folded synthetic protein was obtained, which set the stage for the synthesis of the natural selenoprotein. Moreover, the disulfide pairing mode of the SelF was elucidated through a combination of site-directed mutagenesis and LC-MS study. It provides not only a criterion to judge the viability of the synthetic protein, and more importantly, useful structural insights into the previously unresolved UGGT-binding domain of SelF.

## Introduction

**SCHEME 1:**
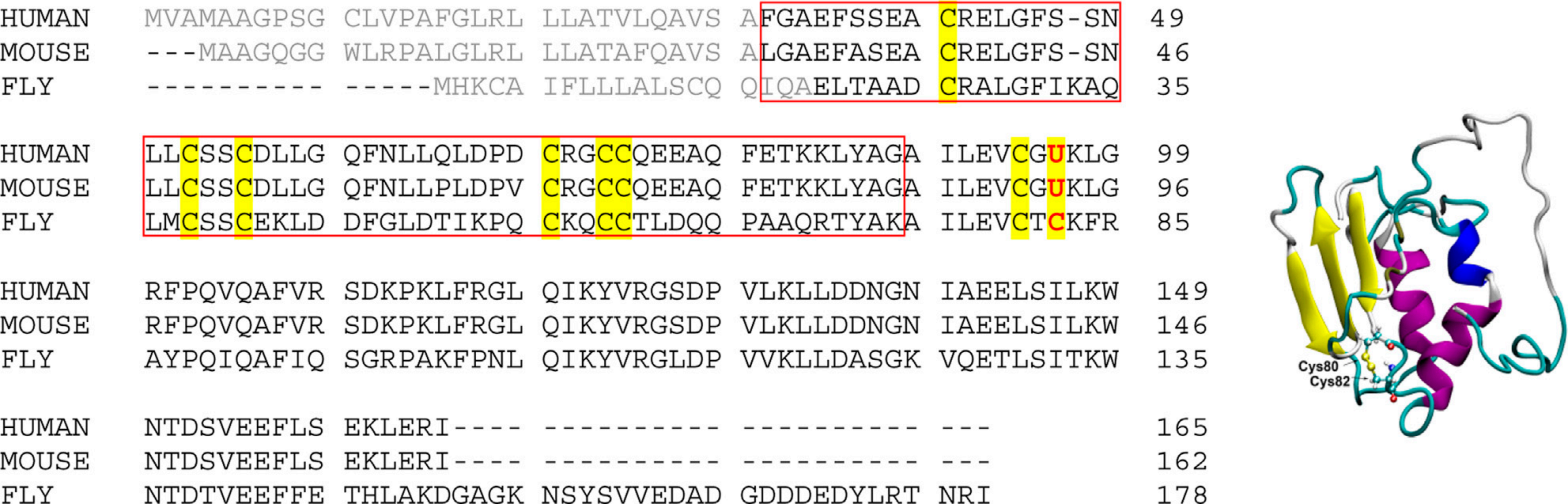
**(Left)** Sequence alignment of Selenoprotein F proteins from representative species. Signal peptide sequences are shown in gray color, and all Cys and Sec residues are highlighted. The UGGT-binding domain is boxed (UniProt ID: O60613, Q9ERR7 and Q9VVJ7 for Sep15 from human, mouse and fruit fly, respectively); **(Right)** NMR structure of the fruit fly Sep15, with only the C-terminal Trx-like domain (61–178) resolved and shown (PDB ID, 2A4H). The disulfide bond is shown in ball and stick representation.

Selenoprotein F (SelF or Selenof), also called the 15-kDa protein (Sep15), is a selenocysteine (Sec, U) containing eukaryotic protein localized to the endoplasmic reticulum (ER) (Gladyshev et al., 1998). As shown in Scheme 1 (gray color), the N-terminal signal peptide was supposed to lead the expressed SelF to the ER, which is cleaved to form the mature protein (Korotkov et al., 2001; Korotkov et al., 2002). While lacking a typical ER retention peptide sequence, the highly conversed Cys-rich domain of SelF is believed to the key for its localization. This domain is able to bind the UDP-glucose:glycoprotein glucosyltransferase (UGGT)—a large chaperone protein in the ER, it is thus also called the UGGT binding domain (Labunskyy et al., 2005). Further, the C-terminal domain of SelF is identified as a thioredoxin (Trx)-like domain, and the CXU/C redox motif (Scheme 1, left) located in a dynamic loop implicates the thiol-disulfide oxidoreductase activity in SelF (Ferguson et al., 2006). As such, SelF is able to play a role in the quality control of the ER (Labunskyy et al., 2007; Labunskyy et al., 2009), but the exact physiological role of the family of protein is yet to be elucidated. A major issue in the study of selenoproteins in general is the lack of reliable recombinant expression techniques, thus most of their biological functions are characterized indirectly, e.g. the knockout assays used in SelF (Joubert et al., 2011; Tsuji et al., 2012; Yim et al., 2018; Zheng et al., 2020). Most of the *in vitro* studies of selenoproteins are thus carried out with the Sec-to-Cys homologue proteins. In this context, the only available structure in the SelF family is reported for a fruit fly Sep15, which contains no Sec residue (Scheme 1, right) (Ferguson et al., 2006), and in this case the two Cys residues (Cys80 and Cys82) in the so-called CXC motif forms a disulfide bond. Unfortunately, the Cys-rich UGGT-binding domain in this structure is too flexible to be resolved in the NMR structure, as such the disulfide pairing mode in this domain is currently not known.

Chemical protein synthesis, powered by chemo-selective peptide ligation reactions, has produced almost hundreds of synthetic proteins which conveniently incorporate non-canonical amino acids, among many other purposes (Agouridas et al., 2019; Tan et al., 2020). Although the chemical synthesis of selenoproteins, like SelM and SelW, has been reported (Dery et al., 2017), the synthesis of SelF is, however, supposed to be more challenge as it contains six other Cys residues in the UGGT binding domain, which certainly complicates the refolding process. Moreover, as mentioned, the disulfide pairing mode is currently unknown, making it difficult to determine whether the synthetic SelF is properly folded.

As part of our ongoing research towards the synthesis of SelF, we document herein the chemical synthesis of its Sec-to-Cys homologue—SelF(U65C). The highly optimized synthetic route as well as the refolding strategy developed in the current work would guide the synthesis of the native SelF protein. Further, besides serving a criterion to judge the viability of the synthetic protein, the disulfide pairing mode of SelF(U65C) also provides useful structural insights into the previously unresolved UGGT-binding domain, and it is thus the aim of the current work to elucidate this key information.

## Materials and Methods

### General Reagents and Methods

Commercially available materials were obtained from Adamas, Energy Chemicals, or Sigma-Aldrich. Standard Fmoc-amino acids, 2-chlorotrityl chloride (2-Cl-(Trt)-Cl) resin, 1-hydroxybenzotriazole (HOBt), and 2-(1 h-benzotriazole-1-yl)-1,1,3,3-tetramethyluronium tetrafluoroborate (TBTU) were purchased from GL Biochem (Shanghai). The reagents acetylacetone (acac) was purchased from Aladdin. Ethylene diamine tetraacetic acid (EDTA) and 2,2′-(ethylenedioxy)diethanethiol (DODT) were purchased from TCI. 2,2′-azobis [2-(2-imidazolin-2-yl) propane] dihydrochloride (VA-044) and silver acetate (AgOAc) were obtained from J&K Scientific and innochem, respectively. *N*,*N*-diisopropylethylamine (DIPEA), *N*,*N*′-diisopropylcarbodiimide (DIC), dithiothreitol (DTT), tris(2-carboxyethyl)phosphine (TCEP), trifluoroacetic acid (TFA), 2-methyl-2-propanethiol (t-BuSH), and guanidine hydrochloride (Gn·HCl) were obtained from Adamas. Methoxylamine (CH_3_ONH_2_·HCl) and triisopropylsilane (TIPS) were purchased from Energy Chemicals. L-arginine hydrochloride (Arg·HCl), tris(hydroxymethyl)aminomethane (Tris), glutathione reduced (GSH) and glutathione oxidized (GSSG) were purchased from Sangon. The reagents *N*,*N*-dimethylformamide (DMF) and dichloromethane (DCM) were purchased from GHTCH (Guangdong). 1,8-diazabicyclo [5.4.0]undec-7-ene (DBU) and 4-mercaptophenylacetic acid (MPAA) were purchased from Alfa Aesar. His_6_-Ulp1 was recombinantly expressed using a reported procedure (Malakhov et al., 2004). Acetonitrile (MeCN) used in analytical HPLC and preparative HPLC was obtained from Fisher and Sigma-Aldrich, respectively. Analytical HPLC (Agilent 1,260) was performed on a Phenomenex Jupiter C4 column (4.6 × 250 mm, 300 Å, 5 μm particle size) running at a flow rate of 1 ml/min with UV detection at 214 and 254 nm. Semi-preparative HPLC (Shimadzu AR-20) was performed using a Waters XBridge® peptide BEH C18 OBD™ Prep column, 300 Å, 5 μm, 10 × 250 mm) running at a flow rate of 4.7 ml/min with UV detection at 214 and 254 nm. Preparative HPLC (Ruihe® Tech) was performed using a Welch Ultimate XB-C4 column Prep column, 300 Å, 5 μm, 30 × 250 mm) running at a flow rate of 40 ml/min with UV detection at 214 and 254 nm. Solvent A: 0.1% TFA in water; Solvent B: 0.1% TFA in MeCN. LC-MS was performed on an Agilent LC/MSD (ESI) system on ACE 5 C4 column (150 × 4.6 mm). MALDI-TOF mass spectra (MALDI-8020, Shimadzu) were obtained in the linear positive mode using a matrix of 10 mg/ml α-Cyano-4-hydroxycinnamic acid (HCCA) in water/MeCN (1: 1, v/v) with 0.1% TFA.

### General Procedures for Peptide Synthesis

#### Preloading of 2-Cl-(Trt)-NHNH_2_ Resin

2-Cl-(Trt)-Cl (0.9 mmol/g, 1 g) was swollen in DMF for 20 min and then washed with DMF (2 × 5 ml), DCM (2 × 5 ml), and DMF (2 × 5 ml). The resin was treated with freshly prepared 5% hydrazine monohydrate in DMF (2 × 20 ml) for 30 min and then washed with DMF (2 × 5 ml), DCM (2 × 5 ml), and DMF (2 × 5 ml). The resin was treated with freshly prepared 5% MeOH in DMF (20 ml) for 10 min and then washed with DMF (2 × 5 ml), DCM (2 × 5 ml), and DMF (2 × 5 ml). DIPEA (1.2 mmol) was added to a solution of Fmoc-AA-OH (0.6 mmol) and TBTU (0.6 mmol) in DMF (5 ml). After 2 min of pre-activation, the mixture was added to the resin, which was then shaken for 2 h at 25°C. The resin was washed with DMF (2 × 5 ml), DCM (2 × 5 ml), and DMF (2 × 5 ml), and then capped with 20% acetic anhydride in DMF (10 ml) for 20 min, and washed with DMF (2 × 5 ml), DCM (2 × 5 ml), and DMF (2 × 5 ml) again (Zheng et al., 2013).

#### Estimation of Amino Acid Loading

The resin (10 mg) loaded with the first amino acid was treated with 2% DBU/DMF (2 ml) for 30 min at 25°C to remove the Fmoc group. The deprotection solution (2 ml) was diluted to 10 ml with MeCN, and then 0.8 ml was further diluted to 10 ml with MeCN. The UV absorbance of the resulting piperidine-fulvene adduct solution was measured (*λ* = 304 nm) to estimate the amino acid loading on the resin.

#### Fmoc Deprotection

The resin was treated with 20% piperidine in DMF (5 ml, 2 × 10 min) at 25°C and then washed with DMF (2 × 5 ml), DCM (2 × 5 ml), and DMF (2 × 5 ml).

#### Coupling of General Amino Acids

Peptides were synthesized on a CS Bio 136XT synthesizer using Fmoc solid phase peptide synthesis (SPPS) chemistry. The following Fmoc amino acids with side-chain protecting groups were used: Fmoc-Ala-OH, Fmoc-Arg (Pbf)-OH, Fmoc-Asn(Trt)-OH, Fmoc-Asp(OtBu)-OH, Fmoc-Gln(Trt)-OH, Fmoc-Glu (OtBu)-OH, Fmoc-Gly-OH, Fmoc-Ile-OH, Fmoc-Leu-OH, Fmoc-Lys(Boc)-OH, Fmoc-Phe-OH, Fmoc-Pro-OH, Fmoc-Ser(tBu)-OH, Fmoc-Thr(tBu)-OH, Fmoc-Tyr(tBu)-OH, Fmoc-Val-OH. SPPS was performed on 2-Cl-(Trt)-Cl resins. Fmoc deprotections were performed with 20% piperidine in DMF (10 min × 2). Couplings were performed with Fmoc amino acid (4.0 equiv to resin substitution), TBTU (3.9 equiv) and DIPEA (8.0 equiv) in DMF for 60 min (45°C). After coupling, unreacted free amine was capped by treatment with 20% acetic anhydride in DMF and then washed with DMF (2 × 5 ml), DCM (2 × 5 ml), and DMF (2 × 5 ml).

#### Coupling of Fmoc-Cys(Trt)-OH or Fmoc-Cys(Acm)-OH

A solution of Fmoc-Cys(Trt)-OH or Fmoc-Cys(Acm)-OH (4 equiv), HOBT (3.9 equiv), and DIC (8 equiv) in DMF was added to the resin. The reaction was shaken for 1 h at 45°C. After coupling, unreacted free amine was capped by treatment with 20% acetic anhydride in DMF and then washed with DMF (2 × 5 ml), DCM (2 × 5 ml), and DMF (2 × 5 ml).

#### Cleavage of the Crude Peptide

The dried resin was treated with TFA/H_2_O/TIPS (95:2.5:2.5 v/v/v), or TFA/H_2_O/DODT (95:2.5:2.5 v/v/v) (2–3 ml per 100 mg of resin) and shaken for 2 h a t 25°C. After filtration, the filtrate was concentrated by blowing with a gentle flow of N_2_. Add the precooled diethyl ether to precipitate crude peptides. The resulted suspension was centrifuged (8,000 rpm, 5 min, 4°C), and the ether layer was decanted. Air-dry the peptide product in the open centrifuge tube for about 30 min. The targeted crude peptide was obtained as the solid.

### Synthesis of Thioester Fragment 1

SelF(1–41)-MPAA **(1)** was synthesized on 2-Cl-(Trt)-Cl resin (theoretical loading: 0.9 mmol/g) using Fmoc-Gly-OH with 0.4 mmol/g loading and elongated according to standard Fmoc-SPPS protocols highlighted in *Preloading of 2-Cl-(Trt)-NHNH_2_ Resin, Estimation of Amino Acid Loading, Fmoc Deprotection, Coupling of General Amino Acids, and Coupling of Fmoc-Cys(Trt)-OH or Fmoc-Cys(Acm)-OH* to afford resin-bound peptide. The peptide was cleaved using TFA/H_2_O/DODT (95:2.5:2.5 v/v/v) for 2 h, and according to *Cleavage of the Crude Peptide* to acquire the crude peptide SelF(1–41)-NHNH_2_ (**1a)**. The crude peptide **1a** (400 mg, assumed 100% purity) was dissolved to 40 mg/ml in 6 M Gn·HCl, 0.2 M Na_2_HPO_4_, pH 3.0, with 10 equiv MPAA, 2.5 equiv acac (from a 0.1 M stock in water) were added to the mixture, and the reaction mixture was stirred for 10 h to form thioester fragment SelF(1–41)-MPAA (**1)**. The mixture was centrifuged (8,000 rpm, 5 min, 4°C), filtered and purified by preparative HPLC at 25°C with a gradient of 25–70% MeCN (with 0.1% TFA) in 25 min to obtain 8 mg of segment **1** (1 g resin; 2.5%). Overall, 18 mg of segment **1** was obtained. The purity and exact mass of the peptide was confirmed using analytical HPLC and ESI-MS, respectively (Supplementary Figure 1).

### Synthesis of Thioester Fragment 2

SelF(42–74)-MPAA (**2)** was synthesized on 2-Cl-(Trt)-Cl resin (theoretical loading: 0.9 mmol/g) using Fmoc-Ala-OH with 0.35 mmol/g loading. Through a similar procedure described in *Synthesis of Thioester Fragment 1* and a preparative HPLC at 25°C with a gradient of 25–50% MeCN (with 0.1% TFA) in 25 min, 25 mg of segment **2** was obtained (1 g resin; 7.4%). Overall, 75 mg of segment **2** was obtained. The purity and exact mass of the peptide was confirmed using analytical HPLC and ESI-MS, respectively (Supplementary Figure 2).

### Expression of His_6_-Sumo-SelF(75-134) and Its Enzymatic Cleavage

The gene encoding for the His_6_-SUMO-SelF(75–134) fusion protein was synthesized and codon-optimized for *E. Coli* expression (GenScript Inc., Nanjing). The synthetic gene was cloned into the pET-30a expression vector using the *Nde*I/*Eco*RI restriction sites. The plasmid was firstly transformed into BL21 (DE3) *E. coli* cells chemically. An overnight culture of the cells harboring an expression vector was inoculated (1:50 dilution) in a 4 L flask containing 30 µg/ml Kanamycin in 2 L LB at 37°C. After reaching an OD_600_ of 0.6–0.8 overexpression of His_6_-SUMO-SelF (75–134) was induced by the addition of 2 ml 1 M IPTG stock solution (final conc. 1 mM) at 37°C for 4 h. Cells were harvested by centrifugation (8,000 rpm, 4°C, 15 min). Typically, 6 g cells were resuspended in 50 ml of cell lysis buffer and lysed by ultrasonication (30–40% power, 3 s on 5 s off, 25 min). The crude lysate was centrifuged (16,000 rpm, 4°C, 20 min) and the supernatant was discarded. The precipitate was stirred at 4°C overnight with 10 ml of Ni-NTA binding buffer to extract His_6_-SUMO-SelF(75–134). The precipitate was removed by centrifugation (16,000 rpm, 30 min, 4°C, 5 cycles) and the supernatant applied to a HisTrap™ FF column (5 ml) at 2 ml/min with a AKTA pure chromatography system. Absorption was monitored at 280 nm. The column was washed with 20 ml of Ni-NTA binding buffer (see Supplementary Material for details). His_6_-SUMO-SelF(75–134) was eluted with 25 ml of Ni-NTA eluting buffer (see Supplementary Material for details) in fractions of 5 ml. The 10 ml fractions with A280 > 0.1 (Supplementary Figure 3A) was gradually dialyzed to the 0.5 L refolding buffer (see Supplementary Material for details) to complete the refolding of SUMO domain. 400 µL of a stock solution of His_6_-Ulp (A280 = 0.5) were added to the folded His_6_-SUMO-SelF(75–134) **(3a)** (10 ml, A280 = 1.8) (Ulp1: protein = 2:50, v/v) and the reaction was incubated for 2 h at 30°C. Equal volume of the buffer containing 6 M Gn·HCl, 200 mM Na_2_HPO_4_, 200 mM CH_3_ONH_2_·HCl, 10 mM TCEP (pH 3), was added to the mixture and the pH was adjusted to 4.0 to remove any C-terminal cyclized byproduct. After overnight incubation, the mixture was centrifuged (8,000 rpm, 5 min, 4°C), filtered and purified by preparative HPLC at 25°C with a gradient of 25–48% MeCN (with 0.1% TFA) in 20 min to obtain 13 mg of SelF(75–134) (**3**) (6.5 mg/L LB) (Supplementary Figure 3C). The purity and exact mass of the peptide was confirmed using analytical HPLC and ESI-MS, respectively (Supplementary Figures 3C-c,D).

### 1^st^ Ligation, Desulfurization and Acm Deprotection

#### 1^st^ Ligation and Desulfurization in One-Pot (on a 5 μmol Scale)

The peptide-thioester **2** (6.5 μmol, 1.3 equiv, 26.4 mg) and Cys-peptide **3** (5 μmol, 1 equiv, 34.8 mg) was dissolved in 2.5 ml of ligation buffer of 6 M Gn·HCl and 2.5 M imidazole with 10 mM TCEP at pH 6.5. The solution was incubated at RT for 1 h (confirmed by LC-MS monitoring). Then, to the ligation reaction mixture was added 295 mg TCEP (final conc. 200 mM), t-BuSH (260 μL, 5%, v/v), and an aqueous solution (2.5 ml) of 0.1 M VA-044 (50 equiv, 250 μmol), and the solution (final pH 6.5) was incubated at 42°C (the reaction was monitored by LC-MS). After the desulfurization was completed (24 h), the mixture was centrifuged (8,000 rpm, 5 min, 4°C), filtered and purified by preparative HPLC at 25°C with a gradient of 25–55% MeCN (with 0.1% TFA) in 30 min to collect the desired fractions and immediately lyophilized, affording the desired protein **4** as a white amorphous powder (29.1 mg, 53.8% isolated yield over two steps). The purity and exact mass of the peptide was confirmed using analytical HPLC and ESI-MS, respectively (Supplementary Figures 4A-g,C).

#### Acm Deprotection (on a 1 μmol Scale)

The peptide **4** (1 μmol, 1.0 equiv, 10.8 mg) was dissolved in a 50% aq. acetic acid (5 ml) containing 1% AgOAc, and the mixture was stirred at 50°C for 2 h in the dark. Then 154.2 mg DTT was added to the mixture, and the formed precipitate was separated by centrifugation. The precipitate was repeatedly washed with 6 M Gn·HCl solution and the combined supernatant (ca. 8 ml) was filtered and purified by preparative HPLC at 25°C with a gradient of 25–55% MeCN (with 0.1% TFA) in 30 min to collect the desired fractions and immediately lyophilized, affording the desired protein **5** as a white amorphous powder (5.17 mg, 49.0%). The purity and exact mass of the peptide was confirmed using analytical HPLC and ESI-MS, respectively (Supplementary Figures 5A-c,B).

### 2^nd^ Ligation and Protein Folding in One-Pot

The peptide-thioester **1** (0.76 μmol, 2 equiv, 3.48 mg) and Cys-peptide **5** (0.38 μmol, 1 equiv, 4 mg) was dissolved in 190 μL of the ligation buffer (6 M Gn·HCl, 200 mM Na_2_HPO_4_, 20 mM TCEP, 50 mM MPAA, pH 6.5). The solution was incubated at RT for 24 h (confirmed by LC-MS monitoring). After the conversion was completed, the ligation reaction mixture was reduced by the addition of 2.86 mg TCEP (final conc. 50 mM) and incubated for 15 min. Then, 200 μL of the ligation mixture (in 50 μL portions) was exchanged into a 150 μL buffer containing 6 M Gn·HCl and 200 mM Na_2_HPO_4_ at pH 6 (Amicon® Ultra-0.5 concentrator, 0.5 ml, 3 K MWCO, 11,000 rpm, 4°C). The resulting ligation mixture (∼600 μL) was added dropwise to a 20 ml refolding buffer (0.4 M Arg·HCl, 0.2 M Tris, 0.1 M (NH_4_)_2_SO_4_, 2 mM EDTA, 0.2 mM GSSG, 1 mM GSH, pH 8.2) (final protein conc. was ∼0.25 mg/ml) at 4°C in 30 min, and was stirred in a refrigerator at 4°C for 12 h. The mixture was centrifuged (8,000 rpm, 5 min, 4°C), filtered and purified by semi-preparative HPLC with a two-step gradient: 10–30% in 5 min, then 30–55% MeCN (with 0.1% TFA) in 30 min to collect the desired fractions and lyophilized, and the desired folded protein SelF(U65C/Q74A) (**7)** as a white amorphous powder (1 mg, 17.6% isolated yield over two steps). The purity and exact mass of the peptide was confirmed using analytical HPLC and ESI-MS, respectively (Supplementary Figures 6A-e,C).

### Expression and Purification of Full-Length SelF(U65C) and Its Cys-To-Ser Variants

The gene coding for the His_6_-SUMO-SelF(U65C) fusion protein was synthesized and codon-optimized for *E. Coli* expression (GenScript Inc., Nanjing). The synthetic gene was cloned into the pET-30a expression vector using the *Nde*I/*Eco*RI restriction sites. Site-directed mutagenesis using the Sit-directed Mutagenesis kit from Sangon (Shanghai) was carried out to afford the two Cys-to-Ser variants, U65C/C42S and U65C/C43S respectively. The resulting expression plasmids were transformed into *E. coli* BL21 (DE3). In a typical protein expression experiment, cells were grown in a 2-L LB medium containing 30 ug/mL of kanamycin at 18°C. Expression was induced after reaching an OD_600_ of 0.6 with 1 M IPTG stock solution (final conc. 0.5 mM) and cells were grown for 20 h at 18°C. The cells were harvested by centrifugation (8,000 rpm, 4°C, 15 min). Typically, 4.5 g of cells were resuspended in 30 ml of cell lysis buffer and lysed by ultrasonication (30–40% power, 3 s on 5 s off, 25 min). The crude lysate was centrifuged (16,000 rpm, 4°C, 30 min, 5 cycles) and the supernatant applied to a HisTrap™ FF column (5 ml) at 2 ml/min with a AKTA pure chromatography system. Absorption was monitored at 280 nm. The column was washed with 20 ml of Ni-NTA binding buffer (see supplementary material for details). The desired proteins were eluted with 25 ml of Ni-NTA eluting buffer (see supplementary material for details) in fractions of 5 ml. The 10 ml fractions with A280 > 0.1 (Supplementary Figures 7, 8, 9A) were collected. Then, 400 µL of a stock solution of His_6_-Ulp (A280 = 0.5) was added to the fusion protein **(8a, 9a or 10a)** (10 ml, A280 = 2.1) (Ulp1/protein = 2/50, v/v) and the reaction was incubated for 2 h at 30°C. An extra alkylation step was carried out for the two variants by adding 100 mg of iodoacetamide (IAM) (10 mg/ml) and incubating for 1 h to protect the free Cys residue with a S-carboxyamidomethyl (CAM) group. The mixture was centrifuged (8,000 rpm, 5 min, 4°C), filtered and purified by preparative HPLC using at 25°C with a gradient of 30–55% MeCN (with 0.1% TFA) in 30 min to obtain the desired proteins: 8–9 mg (4–5 mg/L LB) (Supplementary Figures 7, 8, 9C). The purity and exact mass of the protein was confirmed using analytical HPLC and ESI-MS, respectively (Supplementary Figure 7C–D, 8C–D, 9C–D).

### Circular Dichroism

The secondary structure content of the synthetic SelF(U65C/Q74A) (**7**) was compared to the recombinant SelF(U65C) (**8**) using far-UV CD spectroscopy (200–260 nm). Spectra were recorded on a Chirascan™-Plus Circular Dichroism Spectrometer (Applied Photophysics Ltd., United Kingdom), using a quartz cuvette with a path length of 0.1 cm, and obtained by averaging 3 wavelength scans in 1 nm steps, with a signal averaging time of 1 s and a bandwidth of 1 nm. Each purified protein was dissolved separately in NH_4_HCO_3_ buffer. Measuring conditions: Protein conc.: ∼38 µM; Buffer: 10 mM NH_4_HCO_3_, pH 8. The folding of the expressed proteins was also confirmed by CD spectroscopy, which gave very similar spectrum for each protein (Supplementary Figure 10).

### Enzymatic Digestion

Protein was dissolved in a solution of 25 mM NH_4_HCO_3_ (1 mg/ml, 100 µL). Trypsin (0.5 mg/ml, 10 µL) was firstly added to the mixture to digest the protein (Diemer et al., 2020). The reaction was carried out at 37°C for 2 h. Then, the enzymatic reaction was analyzed by LC-MS directly. Next, chymotrypsin (0.16 mg/ml, 11 µL) was added to the reaction mixture for further digestion. The reaction was carried out at 30°C for 2 h. Finally, the enzymatic reaction was analyzed by LC-MS.

## Results and Discussion

### Synthesis Strategy

**FIGURE 1:**
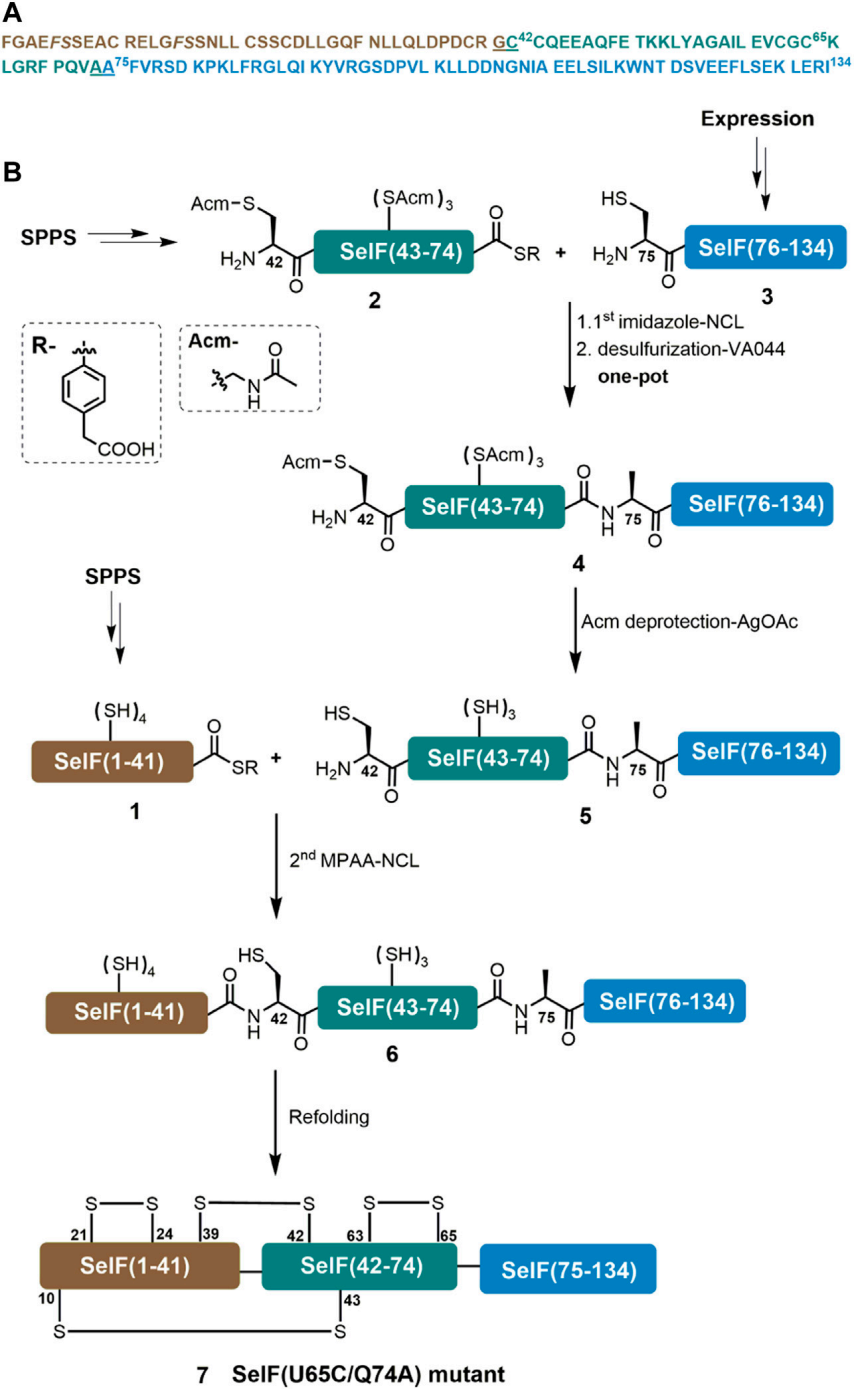
Chemical synthesis of SelF(U65C/Q74A). **(A)** Sequence of the mature form of SelF(U65C/Q74A). The ligation sites are underlined, and the pseudo-proline dipeptide building blocks used during SPPS are shown in italic. **(B)** Synthetic strategy used in the current work.

A major aim of the current work is to establish an efficient synthetic route for the Cys homolog of the mature human SelF protein—which could later be applied for the synthesis of the Sec-containing protein. For this purpose, we initially used a three peptide segments semi-synthetic strategy, in which a short peptide hydrazide segment Cys65–Gln74 was synthesized by SPPS. The remaining two segments could be obtained from recombinant expression. This way we hoped to maximize the synthetic yield of the peptide segments and thus the synthetic protein. An extra C-terminal Cys residue was included in the expression plasmid for the first segment—Phe–Gly64, which was supposed to be able to transform to the corresponding thioester via a reported hydrazinolysis procedure (Adams et al., 2013; Pan et al., 2019). However, the effect of hydrazinolysis is not satisfactory, giving only minor product (data not shown). We thus switched to an alternative strategy where disconnection sites at Gly41–Cys42 and Ala74–Ala75 were adopted. It is noted that for the purpose of applying the peptide hydrazide chemistry, the C-terminal Gln74 residue in the middle segment was mutated to an Ala residue, which should not affect the protein folding and function (vide infra). Here, segments **1** and **2** can be obtained directly from SPPS, while segment **3** can be fused N-terminally to a His_6_-SUMO tag and recombinantly expressed. As shown in Figure 1, the peptide segments were designed to be ligated by native chemical ligation (NCL) (Dawson et al., 1994), and thus an N-terminal Cys residue mutation will be installed in **3** and after its ligation with segment **2**, a desulfurization could lead to the corresponding Ala residue (Wan and Danishefsky, 2007). For this purpose, all Cys sidechains of segment **2** have to be protected with Acm in prior, which after the ligation and desulfurization can be removed. The resulting segment can then be ligated with segment **1** to afford the desired full-length protein **6**.

### Peptide Segments Synthesis

**FIGURE 2:**
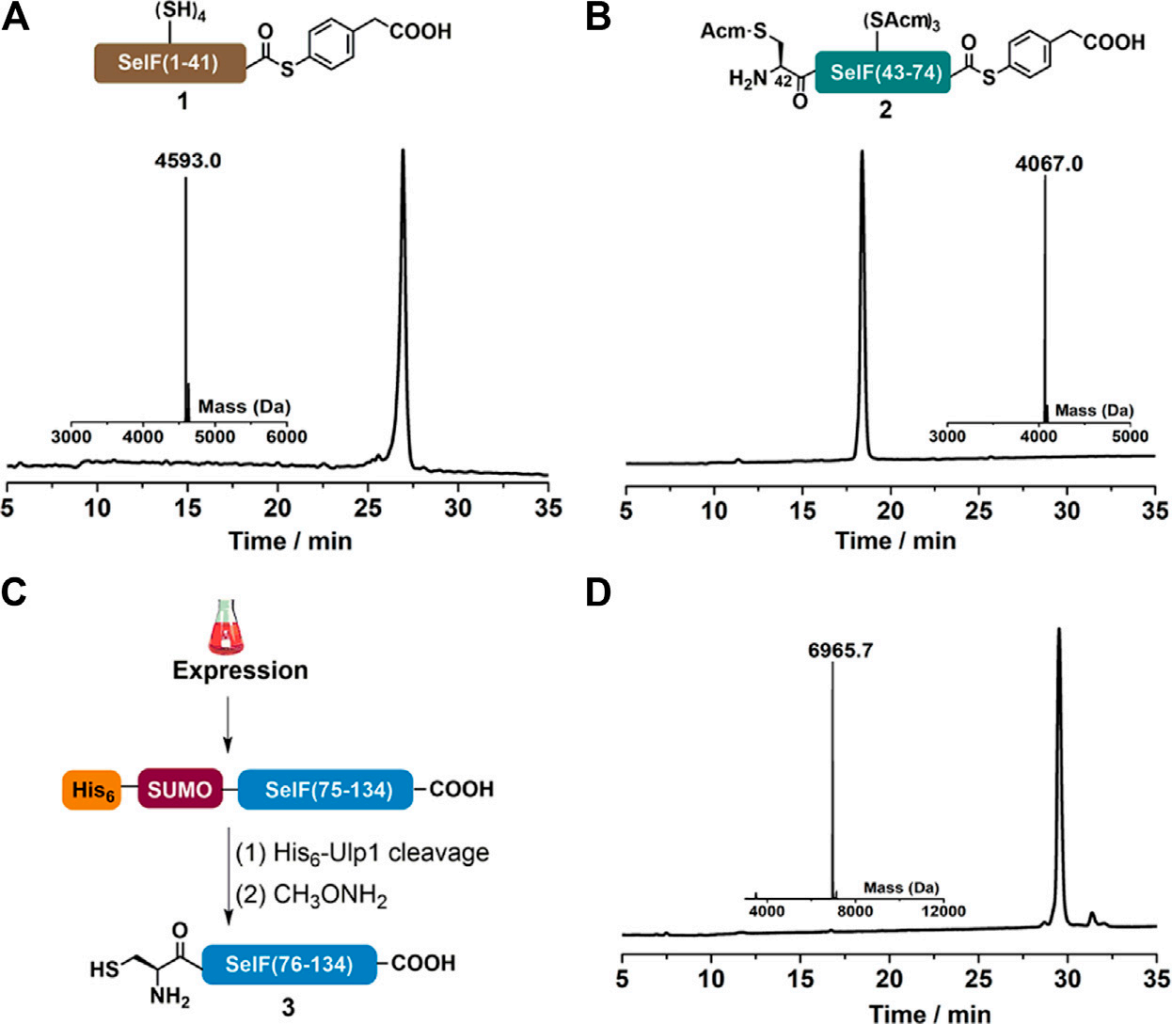
**(A–B)** Analytical HPLC and mass analysis of SelF(1–41)-MPAA **(1)** and SelF(42–74)-MPAA (**2**). **1**: observed mass 4,593.0 Da, calcd 4,593.0 Da (average isotopes); **2**: obsd. mass 4,067.0 Da, calcd. mass 4,066.9 Da (average isotopes). **(C)** Overexpression and Ulp1 cleavage to afford SelF(75–134) (**3**). **(D)** Analytical HPLC and mass analysis of SelF(75–134) (**3**) with the obsd. mass 6,965.7 Da, calcd. mass 6,965.7 Da (average isotopes). Detailed reaction conditions in the Materials and Methods section.

As mentioned, peptide segments **1** and **2** were obtained via SPPS, and C-terminal α-hydrazide was used as thioester precursor. The use of pseudoproline dipeptide Fmoc-Phe-Ser(Ψ^Me,Me^pro)-OH (Wöhr, et al., 1996) proved to be essential for a reasonable yield (Figure 1A). Both segments were cleaved from the resin as peptide hydazides (Fang et al., 2011) and the crude products were transformed into peptide-MPAA thioesters via an acetyl acetone (acac) method before HPLC purification (Figures 2A,B) (Flood et al., 2018). It is noted that NCL with purified peptide thioesters gave better yields than *in situ* method applying peptide hydrides (vide infra) Segment **3** was obtained recombinantly with an N-terminal His_6_-SUMO tag (Figure 2C) (Malakhov et al., 2004; Reif et al., 2014). And after Ulp1-cleavage, the desired segment **3** was obtained with high purity and yield (6.5 mg/L LB, Figure 2D). It is noted that before HPLC purification, the Ulp1-cleavage product was treated with methoxylamine to remove any cyclized thiazolidine byproduct at the N-terminus (Reif et al., 2014).

### 1^st^ Ligation Between Segments 2 and 3

**FIGURE 3:**
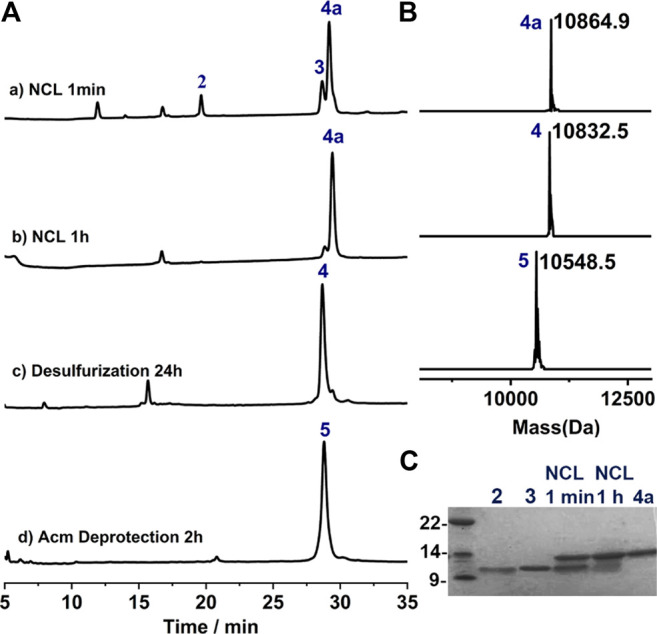
**(A)** Analytical HPLC traces of one-pot NCL-Desulfurization reaction and the Acm group deprotection reaction. **(B)** Mass analysis of ligation reaction product **4a**, **4** and **5**, respectively. **4a**: observed mass 10,864.9 Da, calcd 10,865.6 Da (average isotopes); **4**: obsd. mass 10,832.5 Da, calcd. mass 10,832.6 Da (average isotopes); **5**: obsd. mass 10,548.5 Da, calcd. mass 10,548.5 Da (average isotopes). **(C)** Analytical SDS-PAGE analysis of the ligation reaction.

With all the peptide in hand, we preceded to assemble the full-length protein via NCL. The first NCL was carried between peptide segments **2** and **3**, and with the purpose of applying one-pot desulfurization where the use of MPAA as catalyst is problematic, imidazole was instead used as the catalyst for the ligation (Sakamoto et al., 2016). As shown in Figure 3A,B, the ligation went almost to completion within 1 h, and the reaction progress is better overviewed from the SDS-page analysis (Figure 3C). To this mixture, TCEP and VA-044 were added directly to achieve quantitative desulfurization at Cys75, leading to peptide **4** with excellent isolated yield (29.1 mg, 53.8% over two steps). And after HPLC purification, the Acm protection groups can be conveniently removed using AgOAc (Murar et al., 2020), affording **5** with high isolated yield (14.2 mg, 49.0%, Figure 3A, d).

### 2^nd^ Ligation and Protein Folding

**FIGURE 4:**
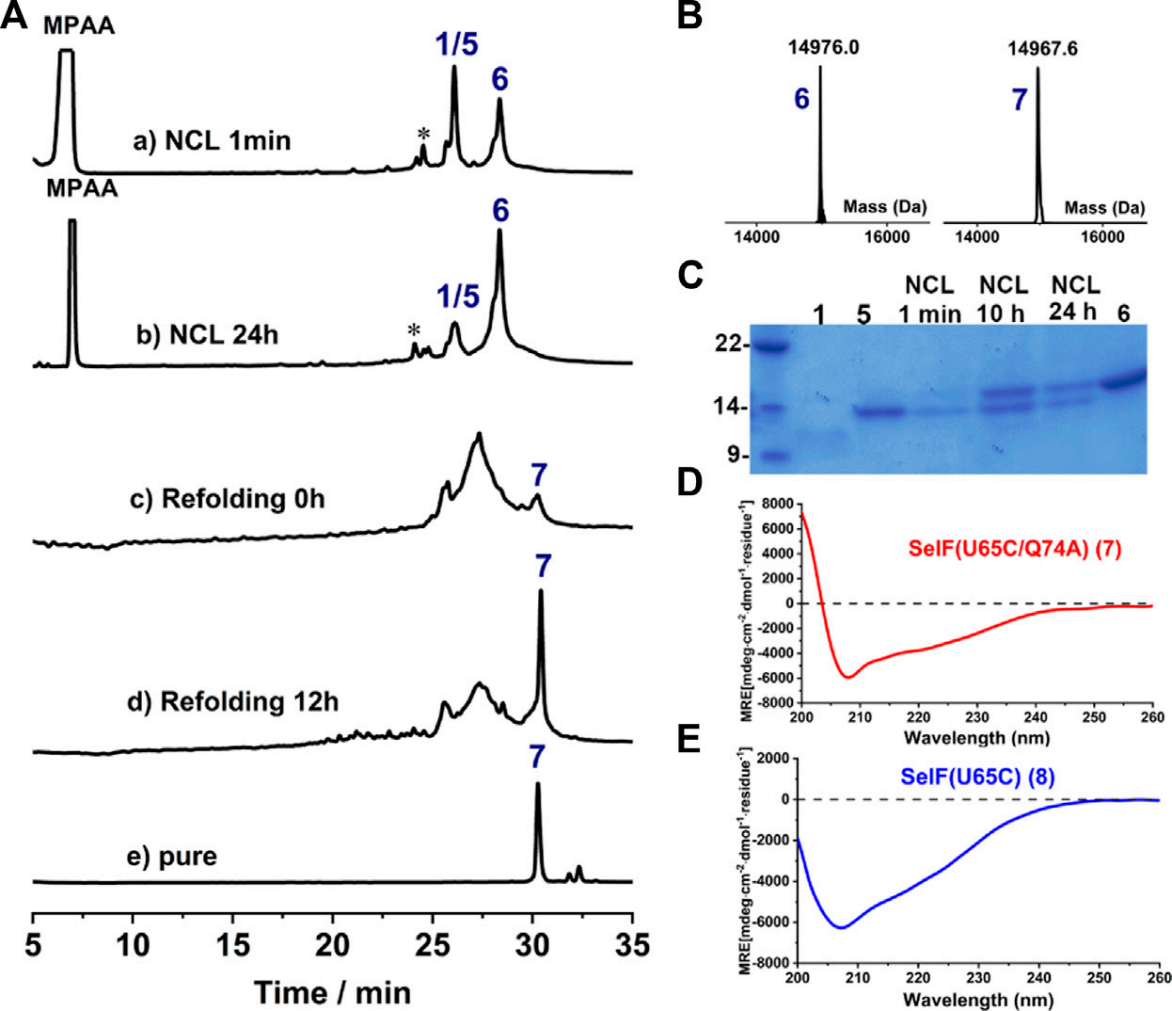
**(A)** Analytical HPLC traces of ligation and refolding reaction of SelF(U65C/Q74A). **(B)** Mass analysis of ligation reaction product **6** and its folded protein **7**, respectively. **6:** obsd. mass 14,976.0 Da, calcd. mass 14,976.2 Da (average isotopes); **7:** obsd. mass 14,967.6 Da, calcd. mass 14,968.2 Da (average isotopes). **(C)** Analytical SDS-PAGE analysis of the ligation reaction. **(D,E)** CD spectra of the fully synthetic SelF(U65C/Q74A) (**7**) **(D)** and the recombinant SelF (U65C) (**8**) **(E)**.

Further, a second ligation between peptide **1** and **5** was carried out to obtain the full-length synthetic protein. In this case, MPAA was added as the catalyst and the ligation proceeded smoothly, albeit with a slow reaction rate compared to the first ligation. The presence of thiolactones (indicated with * in Figure 4A, a-b) resulting from the thiol-exchange between the C-thioester and thiol side-chains in peptide **1** was the key for the slow reaction rate. The ligation was allowed to proceed for 24 h and the resulting solution was directly exchanged into refolding buffer, according to the literature (Vetter et al., 2009) (Figure 4A-C). Gratifyingly, a sharp peak with high intensity in HPLC appeared after 12 h, which as judged from ESI-MS analysis (∼–8 Da compared to the unfolded protein, **6**) corresponds to the folded synthetic protein **7**. Following semi-preparative HPLC purification, **7** was obtained with an isolated yield of 17.6% (3 mg, over two steps, ligation and refolding). Finally, the folded synthetic protein **7** shows identical CD spectrum to the expressed protein counterpart (Figures 4D,E), confirming the correct formation of the 3D structure (Dery et al., 2017).

### Disulfide Pairing Mode Analysis

As mentioned earlier, the disulfide pairing mode of SelF has not been established. The elucidation of such a pairing mode would shed light on its overall structure, especially when the 3D structure of the UGGT binding domain is currently not known. Herein, we identified all disulfide bonds in the SelF(U65C) through a two-step enzymatic digestion of both synthetic and expressed proteins. As shown in Table 1, following trypsin treatment of the recombinant protein SelF(U65C), **8**, a peptide fragment bearing the Cys63–Cys65 disulfide can be clearly observed (Entry 1) (Supplementary Figure 12A), which agrees well with the reported NMR structure of a homolog protein—the fruit fly Sep15 (Ferguson et al., 2006). Further treatment of the peptide mixture with chymotrypsin allows the identification of a peptide fragment containing Cys21–Cys24 (Table 1, Entry 3) (Supplementary Figure 12B). The connectivity mode of the remaining four Cys residues is, however, difficult to resolve as Cys42 and Cys43 are next to each other. In this case, we created two Cys-to-Ser variants, i.e. SelF(U65C/C42S) and SelF(U65C/C43S), and the now-free Cys in each variant was firstly protected with IAM. The resulting proteins—SelF(U65C/C42S)-CAM, **9** and SelF(U65C/C43S)-CAM, **10**—were then processed through a similar sequential trypsin/chymotrypsin digestion, and the remaining two disulfide bonds were established as Cys10–Cys43 and Cys39–Cys42, respectively (Table 1, Entries 4 and 7) (Supplementary Figure 13A, 14B). Importantly, the synthetic protein showed the same disulfide pairing mode as the expressed protein (Supplementary Figure 11, 12), which reassures the viability of the synthetic strategy developed in the current work to provide authentic samples for further biological studies.

**TABLE 1:** Disulfide bond elucidation through the enzymatic digestion of SelF(U65C) (**8**), SelF(U65C/C42S)-(CAM) (**9**) and SelF(U65C/C43S)-(CAM) (**10**).

Entry	Protein variant	Digestion	Sequences	Molecular weight (Da)	
				Obs	Calc
1	SelF(U65C) (**8**)	Trypsin	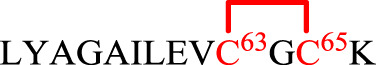	1,336.6	1,336.7
2		Trypsin and chymotrypsin	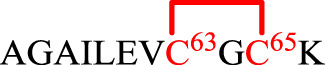	1,060.7	1,060.5
3			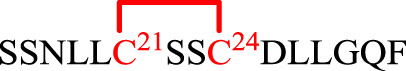	1,583.0	1,583.7
4	SelF(U65C/C42S)-CAM (**9**)	Trypsin	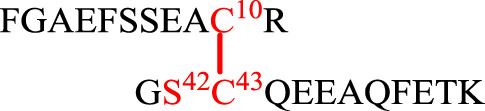	2,557.0	2,556.7
5		Trypsin and chymotrypsin	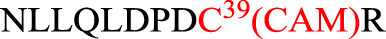	1,243.0	1,242.5
6	SelF(U65C/C43S)-CAM (**10**)	Trypsin	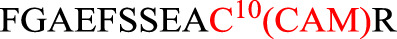	1,259.0	1,259.2
7		Trypsin and chymotrypsin	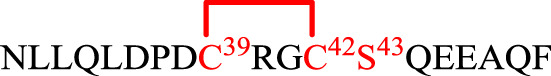	2,180.9	2,181.3

## Conclusion

In summary, we have developed an efficient synthetic strategy affording the Cys homologue of human SelF protein. It highlights the use of two one-pot operations, i.e. the first ligation and desulfurization and the second ligation and refolding, and through this highly optimized strategy, multi-milligram of folded SelF(U65C/Q74A) was obtained. Given that the Sec65 residue is in the middle of the segment **2**, we envision that the synthetic and refolding strategies developed in this study can be directly applied in the chemical synthesis of the native SelF. As such, it would not only provide enough authentic samples for further biological studies, but also set the stage for the synthesis of the selenocysteine-containing protein—SelF. Moreover, the disulfide pairing mode of SelF has been elucidated for the first time, which should provide a good opportunity to understand its unique biological functions, such as its binding to UGGT.

## Data Availability

The datasets presented in this study can be found in online repositories. The names of the repository/repositories and accession number(s) can be found in the article/Supplementary Material.
