# Supplementary material for: Chemical Synthesis of the Sec-To-Cys Homologue of Human Selenoprotein F and Elucidation of Its Disulfide-pairing Mode
Source: Front Chem. 2021 Jul 29;9:735149. doi: 10.3389/fchem.2021.735149 (PMC8360269; doi:10.3389/fchem.2021.735149)
Supplement: Supplementary file 1 [file DataSheet1.pdf]

Chemical synthesis of the Sec-to-Cys homologue of human selenoprotein F and elucidation of its disulfide-pairing mode

Peisi Liao[1], Chunmao He[1*]

[1]School of Chemistry and Chemical Engineering, South China University of Technology, Guangzhou, China

*** Correspondence:**
Chunmao He
hecm@scut.edu.cn

Supplementary Material

1 Supplementary Figures

1.1 Synthesis of thioester fragment 1

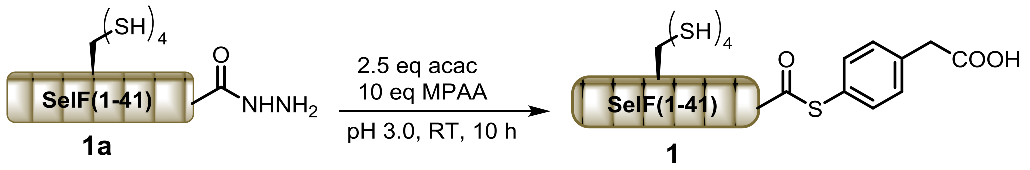

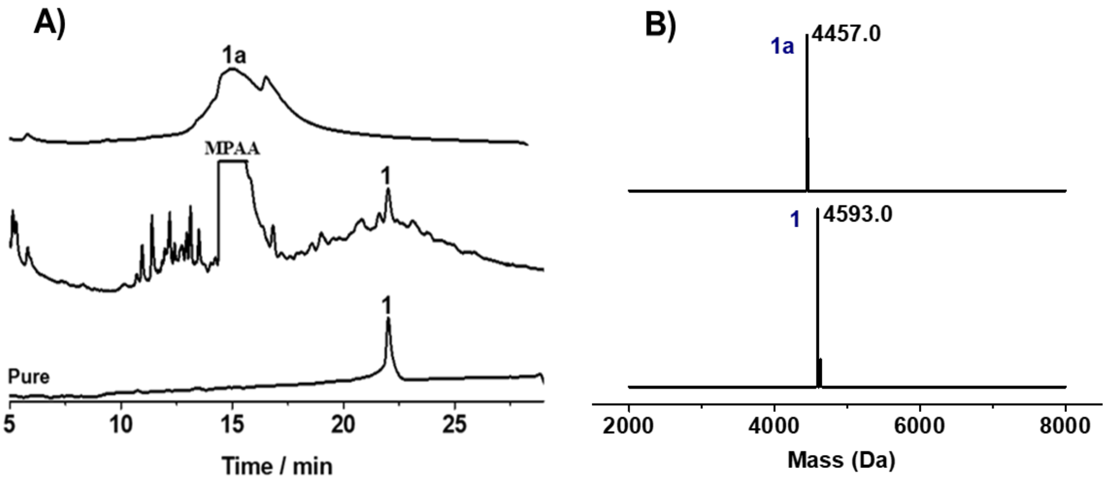

Supplementary Figure 1. (A) Analytic HPLC trace (25 to 70% MeCN (with 0.1% TFA) in 25 min, λ = 214 nm) of crude segment **1a** and purified segment **1.** ESI-MS analysis of **1a** with the observed mass 4457.0 Da, calcd 4457.0 Da (average isotopes). ESI-MS analysis of **1** with the observed mass 4593.0 Da, calcd 4593.0 Da (average isotopes).

1.2 Synthesis of thioester fragment 2

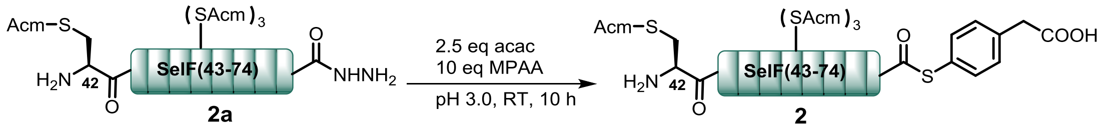

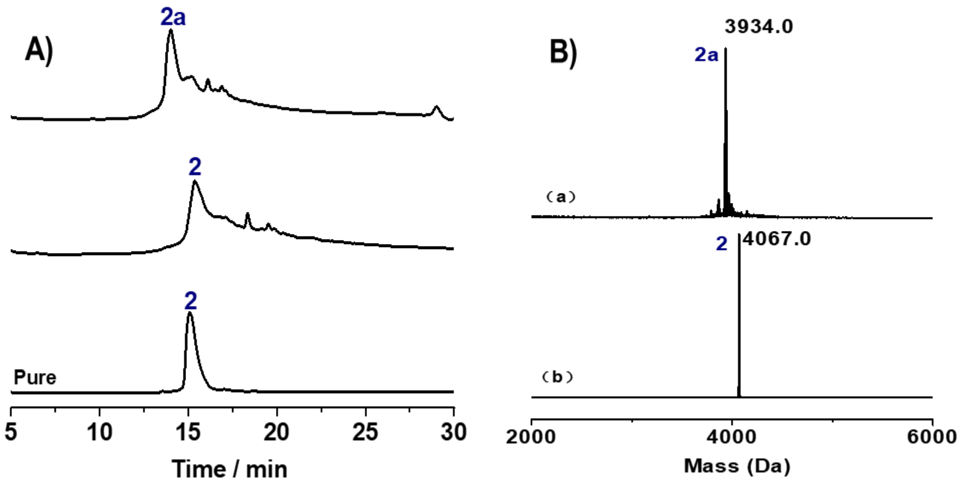

Supplementary Figure 2. (A) Analytic HPLC trace (25 to 55% MeCN (with 0.1% TFA) in 25 min, λ = 214 nm) of crude segment **2a** and purified segment **2.** (B) (a) MALDI-TOF analysis of **2a** with the observed mass 3934.0 Da, calcd 3930.9 Da (average isotopes). (b) ESI-MS analysis of **2** with the observed mass 4067.0 Da, calcd 4066.9 Da (average isotopes).

1.3 Expression of His$_6$-SUMO-SelF(75-134) and Ulp1 cleavage

The vector pET-30a (+)-SelF (75-134) required for the overexpression of the desired His$_6$-SUMO-SelF (75-134) can be obtained from GL Biochem. The cleavage site between Gly and Cys is marked in bold. The full amino acid sequence of His$_6$-SUMO-SelF (75-134) was:

MNWSHPQFEKSSGSSGGHHHHHHGGSGGSGSDSEVNQEAKPEVKPEVKPETHINLKVSDGS
SEIFFKIKKTTPLRRLMEAFAKRQGKEMDSLRFLYDGIRIQADQAPEDLDMEDNDIIEAHREQ
IG**GC**FVRSDKPKLFRGLQIKYVRGSDPVLKLLDDNGNIAEELSILKWNTDSVEEFLSEKLERI

Overexpression and purification of His$_6$-SUMO-SelF(75-134):

LB (2 L): 10 g/L tryptone, 5 g/L yeast extract, 10 g/L NaCl, pH 7

Cell lysis buffer: 0.9% NaCl, pH 7

Extraction buffer: 8 M Urea, 20 mM imidazole, 0.5 M NaCl, 20 mM Tris, pH 8

Ni-NTA binding buffer: 8 M Urea, 20 mM Imidazole, 0.5 M NaCl, 20 mM Tris, pH 8

Ni-NTA eluting buffer: 8M Urea, 250 mM Imidazole, 0.5 M NaCl, 20 mM Tris, pH 8

Refolding buffer: 6 M Urea, 0.15 M NaCl, 20 mM Tris, 10% glycerol, pH 8; 4 M Urea, 0.15 M NaCl, 20 mM Tris, 10% glycerol, pH 8; 2 M Urea, 0.15 M NaCl, 20 mM Tris, 10% glycerol, pH 8; 1 M Urea, 0.15 M NaCl, 20 mM Tris, 10% glycerol, pH 8; 0.15 M NaCl, 20 mM Tris, 10% glycerol, pH 8, these dialysates were changed every 12 hours.

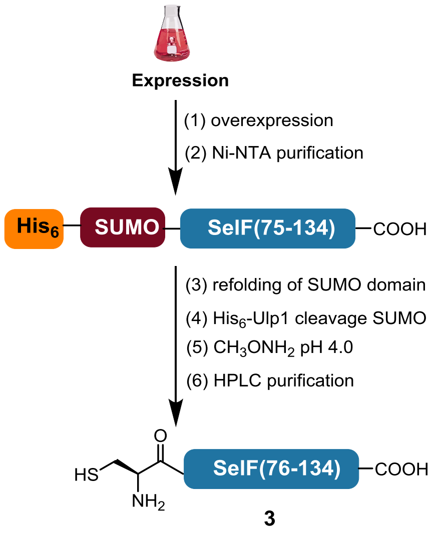

SUMO = Small Ubiquitin-like Modifier
Ni-NTA = Nicker nitrilotriacetic acid complex

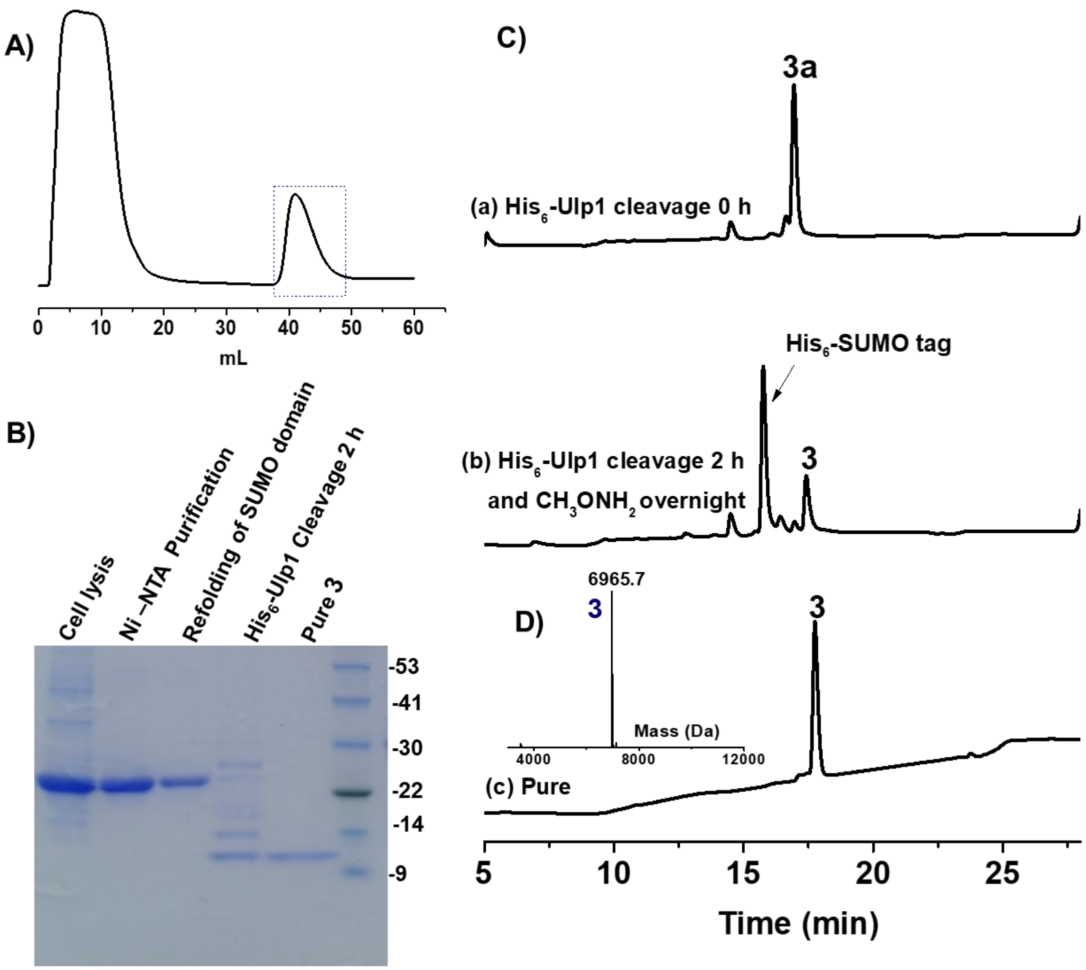

Supplementary Figure 3. (A) Purified by Histrap^{TM} FF column (λ = 280 nm) of His$_6$-SUMO-SelF (75-134)**.** (B) SDS-PAGE of the His$_6$-SUMO-SelF(75-134) cell lysis, Ni-NTA purification and refolding of **3a** followed by cleavage of the His$_6$-SUMO tag. Lane 1: soluble fraction after cell lysis; lane 2: after Ni-NTA purification; lane 3: refolding of **3a**; lane 4: cleavage of the His$_6$-SUMO tag; lane 5: pure SelF(75-134) (**3)**; lane 6: molecular weight standard. (C) Analytic HPLC trace (15 to 65% MeCN (with 0.1% TFA) in 20 min, λ = 214 nm) of 3**a**, the cleavage of the His$_6$-SUMO tag from refolded **3a** and pure **3.** (D) ESI-MS analysis of **3** with the observed mass 6965.7 Da, calcd 6965.7 Da (average isotopes).

1.4 1st ligation and desulfurization in one-pot (on a 5 µmol Scale)

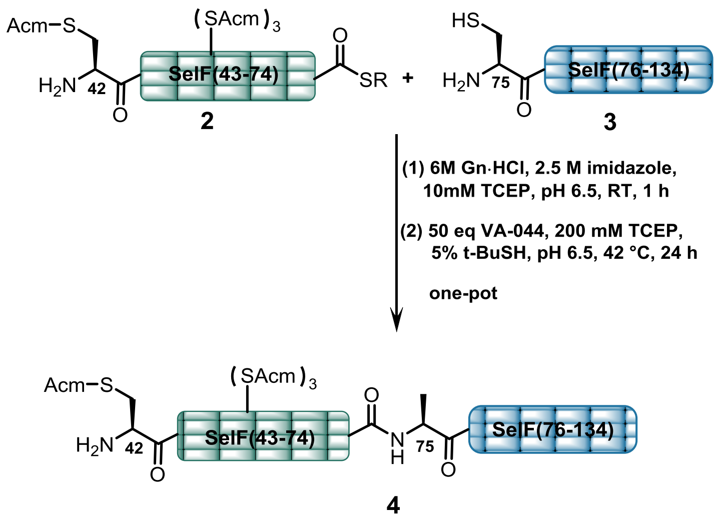

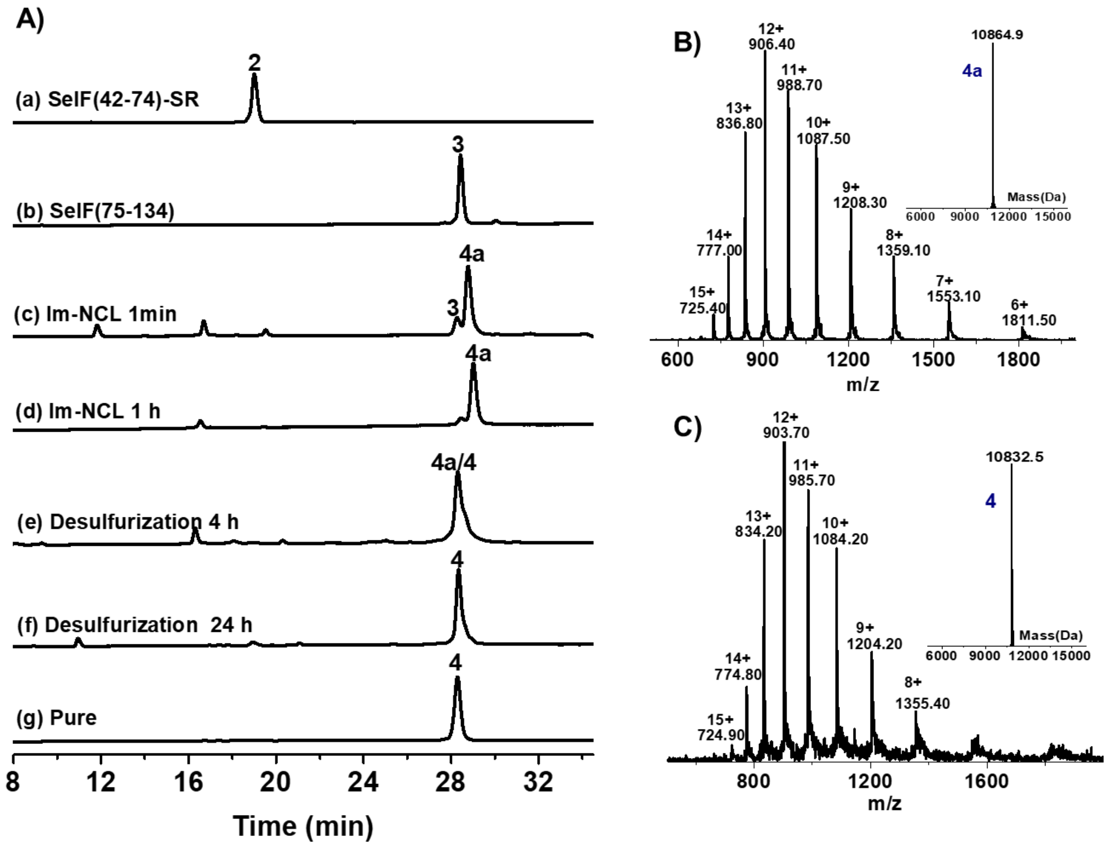

Supplementary Figure 4. (A) Analytic HPLC trace (25 to 55% MeCN (with 0.1% TFA) in 30 min, λ = 214 nm) of imidazole-aided NCL (Im-NCL) and desulfurization (one-pot), and the purified **4**. (B) ESI-MS analysis of **4a** with the observed mass 10864.9 Da, calcd 10865.6 Da (average isotopes) (C) ESI-MS analysis of **4** with the observed mass 10832.5 Da, calcd 10832.6 Da, (average isotopes).

1.5 Acm deprotection (on a 1 μmol Scale)

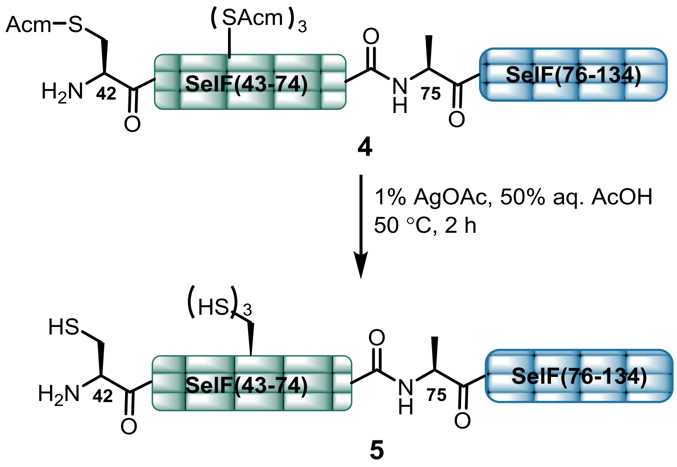

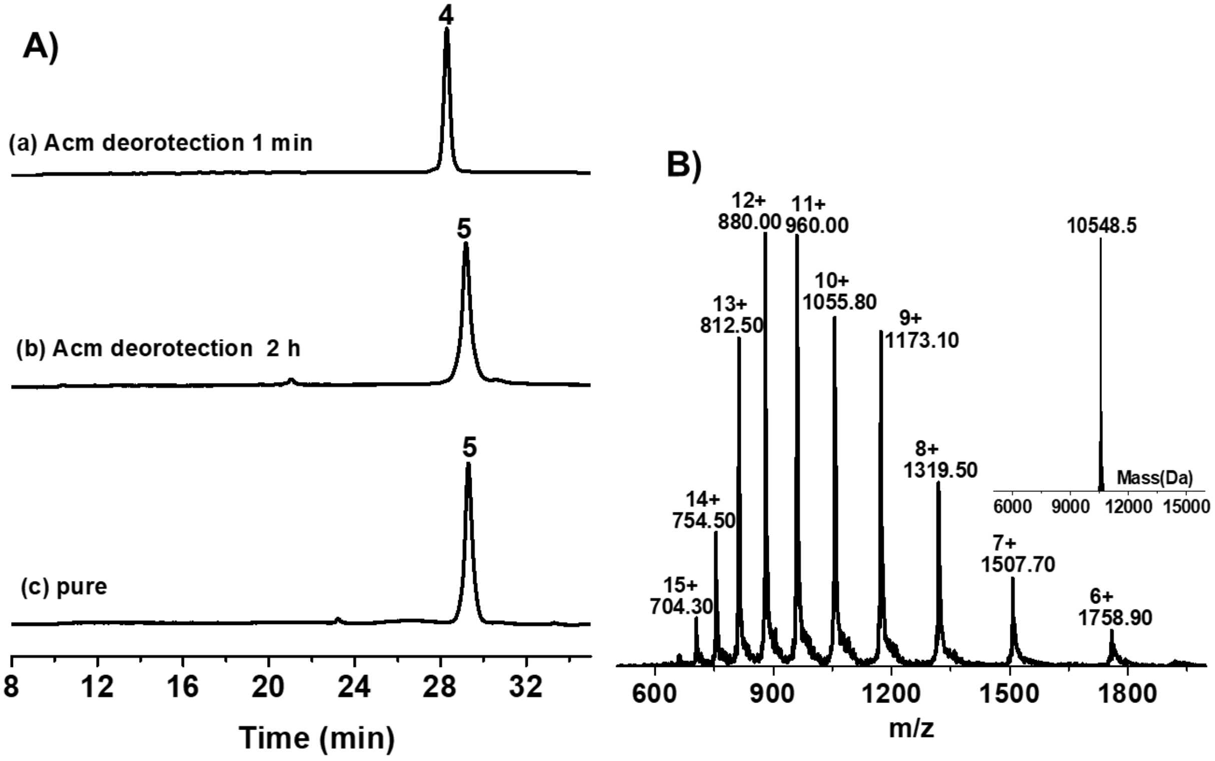

Supplementary Figure 5. (A) Analytic HPLC trace (25 to 55% MeCN (with 0.1% TFA) in 30 min, λ = 214 nm) of Acm deprotection and the purified **5**. (B) ESI-MS analysis of **5** with the observed mass 10548.5 Da, calcd 10548.5 Da (average isotopes).

1.5 2nd ligation and protein folding in one-pot

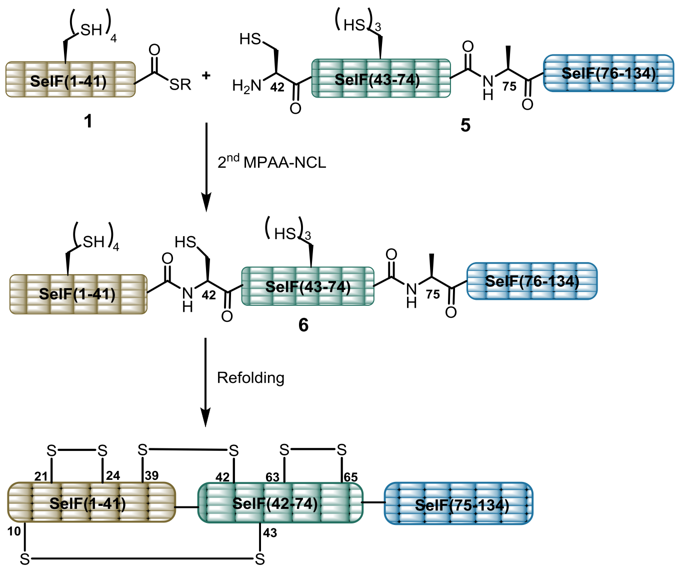

7 SelF(U65C) mutant

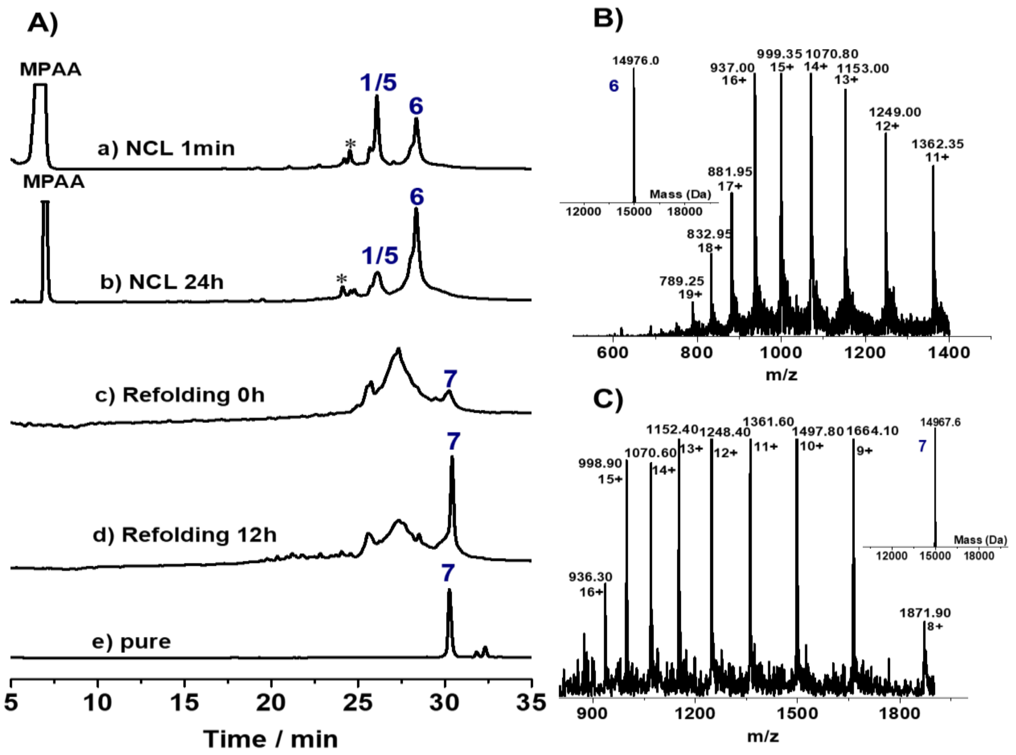

Supplementary Figure 6. (A) Analytical HPLC traces (30 to 55% MeCN (with 0.1% TFA) in 30 min, λ = 214 nm) of ligation and refolding reaction of SelF(U65C/Q74A). (B) ESI-MS analysis of **6** with the observed mass 14976.0 Da, calcd 14976.2 Da (average isotopes). (C) ESI-MS analysis of **7** with the observed mass 14967.6 Da, calcd 14968.2 Da (average isotopes).

1.6 Expression and purification of full-length SelF(U65C) mutant

The vector pET-30a (+)-SelF(U65C) required for the overexpression of the desired His_6-SUMO-SelF(U65C) can be obtained from GL Biochem. The cleavage site between Gly and Phe is marked in bold. The full amino acid sequence of His_6-SUMO-SelF(U65C) was:

MNWSHPQFEKSSGSSGGHHHHHHGGSGGSGSDSEVNQEAKPEVKPEVKPETHINLKVSDGS
SEIFFKIKKTTPLRRLMEAFAKRQGKEMDSLRFLYDGIRIQADQAPEDLDMEDNDIIEAHREQ
IG**GF**GAEFSSEACRELGFSSNLLCSSCDLLGQFNLLQLDPDCRGCCQEEAQFETKKLYAGAIL
EVCGCKLGRFPQVQAFVRSDKPKLFRGLQIKYVRGSDPVLKLLDDNGNIAEELSILKWNTDS
VEEFLSEKLERI

Expression and purification of His_6-SUMO-SelF(U65C):

LB (2 L): 10 g/L tryptone, 5 g/L yeast extract, 10 g/L NaCl, pH 7

cell lysis buffer: 5 mM Imidazole, 0.5 M NaCl, 20 mM Tris, pH 8

Ni-NTA binding buffer: 5 mM Imidazole, 0.5 M NaCl, 20 mM Tris, pH 8

Ni-NTA eluting buffer: 250 mM Imidazole, 0.5 M NaCl, 20 mM Tris, pH 8

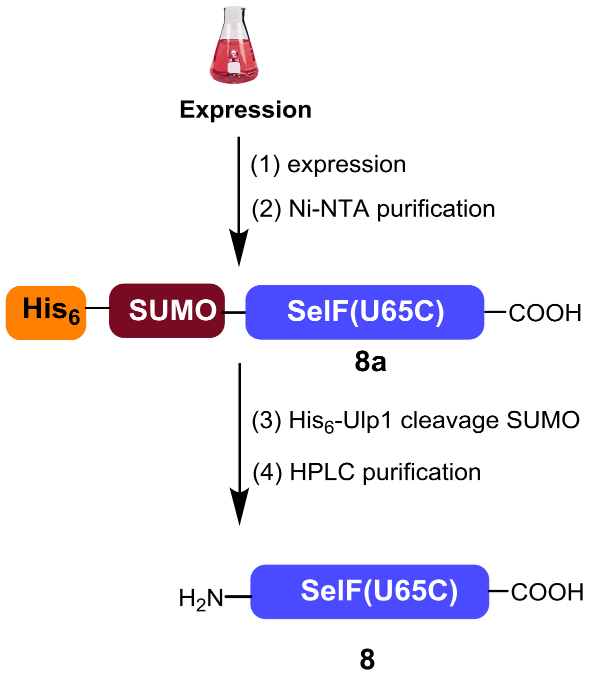

SUMO = Small Ubiquitin-like Modifier
Ni-NTA = Nicker nitrilotriacetic acid complex

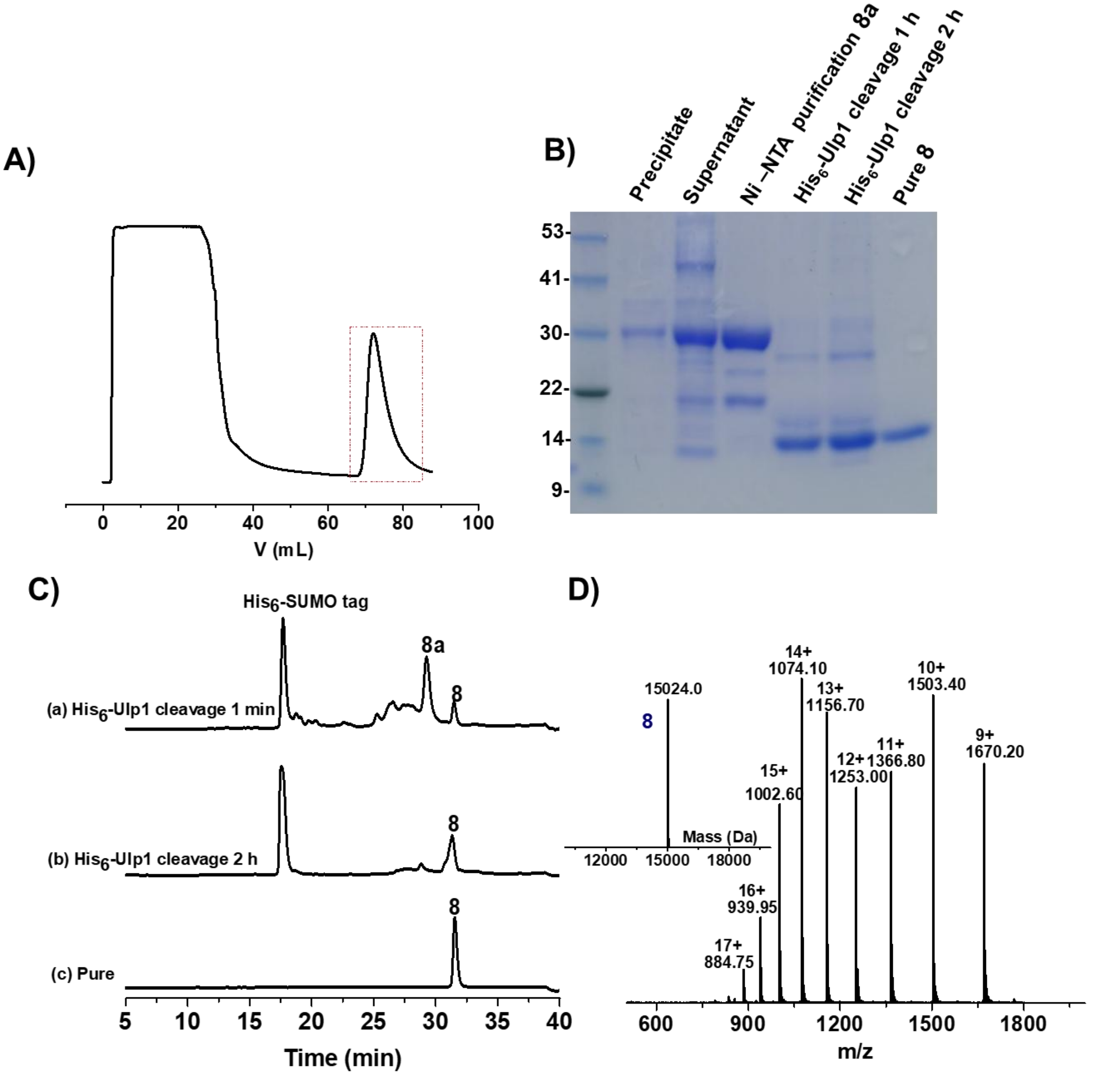

Supplementary Figure 7. (A) Purified by Histrap™ FF column (λ = 280 nm) of His$_6$-SUMO-SelF(U65C) (**8a**). (B) SDS-PAGE of the His$_6$-SUMO-SelF (U65C) cell lysis, Ni-NTA purification and **8a** followed by cleavage of the His$_6$-SUMO tag. lane 1: molecular weight standard. Lane 2: precipitate after cell lysis; Lane 3: soluble fraction after cell lysis; lane 4: after Ni-NTA purification of **8a**; lane 5: cleavage the His$_6$-SUMO tag 1 h; lane 6: cleavage the His$_6$-SUMO tag 2 h; lane 7: pure SelF(U65C) (**8**); (C) Analytic HPLC trace (30 to 55% MeCN (with 0.1% TFA) in 30 min, λ = 214 nm) of **8a**, the cleavage of the His$_6$-SUMO tag from **8a** and pure **8**. (D) ESI-MS analysis of **8** with the observed mass 15024.0 Da, calcd 15025.2 Da.

1.7 Expression and purification of SelF(U65C/C42S)-CAM mutant

The vector pET-30a (+)-SelF (U65C/C42S) required for the expression of the desired His$_6$-SUMO-SelF(U65C/C42S). The cleavage site between Gly and Phe is marked in bold. The full amino acid sequence of His$_6$-SUMO-SelF(U65C/C42S) was:

MNWSHPQFEKSSGSSGGHHHHHHGGSGGSGSDSEVNQEAKPEVKPEVKPETHINLKVSDGS
SEIFFKIKKTTPLRRLMEAFAKRQGKEMDSLRFLYDGIRIQADQAPEDLDMEDNDIIEAHREQ
IG**GF**GAEFSSEACRELGFSSNLLCSSCDLLGQFNLLQLDPDCRGSCQEEAQFETKKLYAGAIL
EVCGCKLGRFPQVQAFVRSDKPKLFRGLQIKYVRGSDPVLKLLDDNGNIAEELSILKWNTDS
VEEFLSEKLERI

Expression and purification of His$_6$-SUMO-SelF(U65C/C42S):

LB (2 L): 10 g/L tryptone, 5 g/L yeast extract, 10 g/L NaCl, pH 7

cell lysis buffer: 5 mM Imidazole, 0.5 M NaCl, 20 mM Tris, pH 8

Ni-NTA binding buffer: 5 mM Imidazole, 0.5 M NaCl, 20 mM Tris, pH 8

Ni-NTA eluting buffer: 250 mM Imidazole, 0.5 M NaCl, 20 mM Tris, pH 8

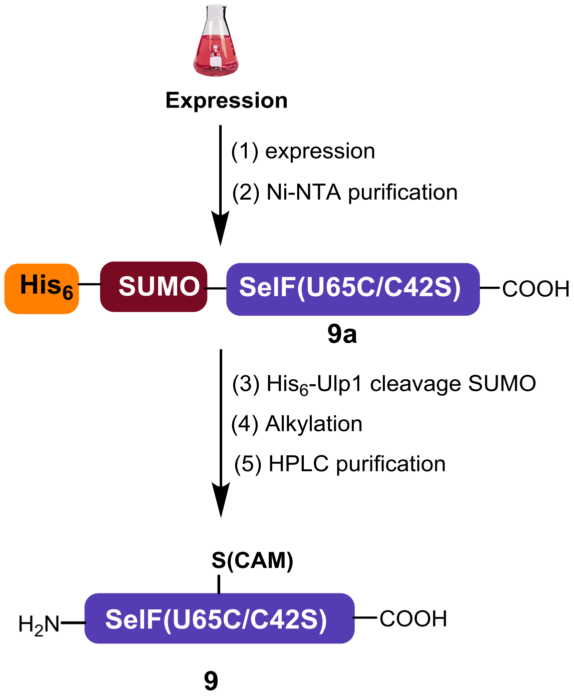

SUMO = Small Ubiquitin-like Modifier
Ni-NTA = Nicker nitrilotriacetic acid complex

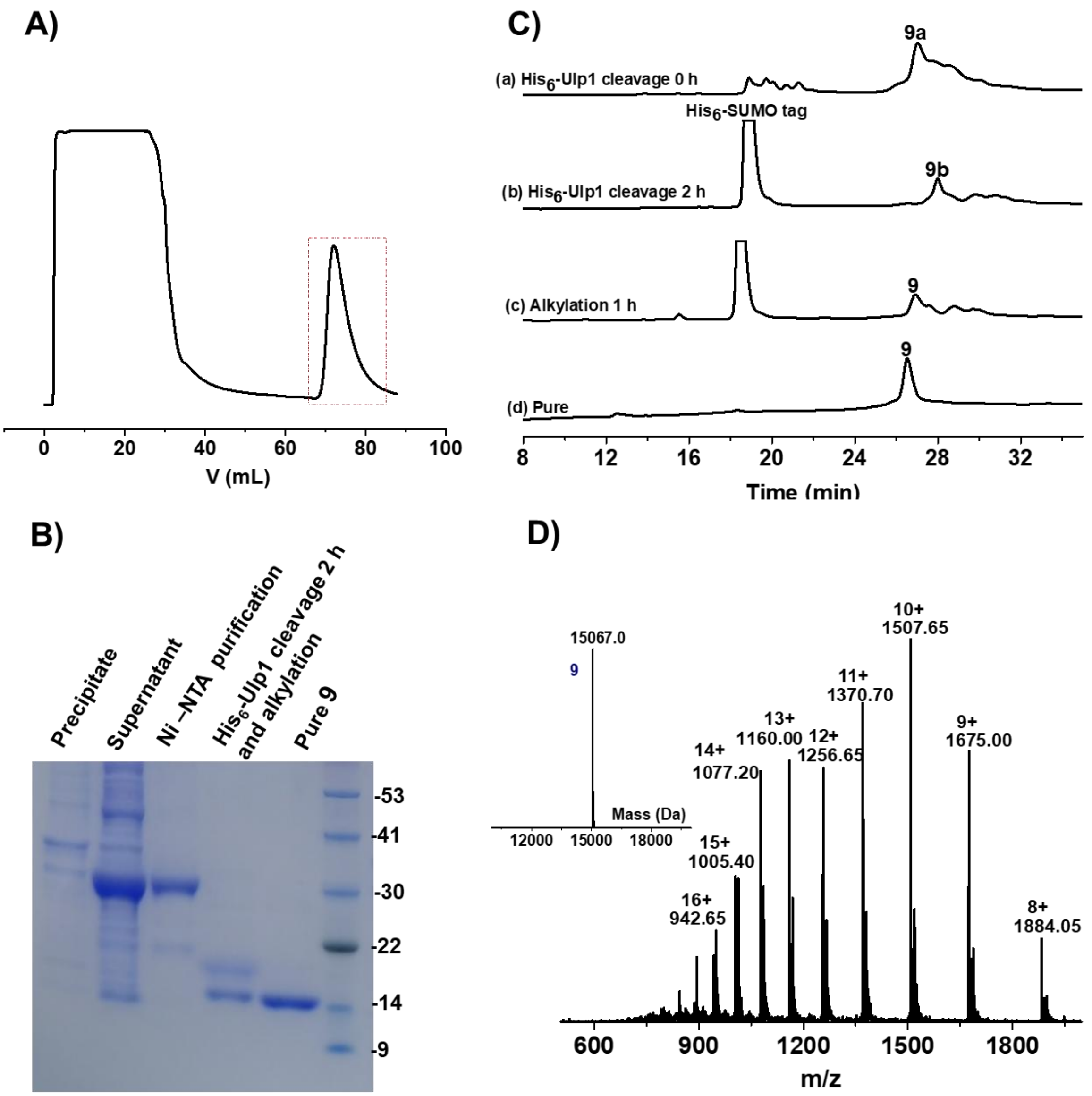

Supplementary Figure 8. (A) Purified by Histrap^{TM} FF column (λ = 280 nm) of His$_6$-SUMO-SelF(U65C/C42S) (**9a**). (B) SDS-PAGE of the His$_6$-SUMO-SelF(U65C/C42S) cell lysis, Ni-NTA purification and **9a** followed by cleavage of the His$_6$-SUMO tag. Lane 1: precipitate after cell lysis; Lane 2: soluble fraction after cell lysis; lane 3: after Ni-NTA purification of **9a**; lane 4: cleavage His$_6$-SUMO tag 2 h and alkylation 1 h; lane 5: pure SelF(U65C/C42S)-CAM (**9**); lane 6: molecular weight standard. (C) Analytic HPLC trace (30 to 55% MeCN (with 0.1% TFA) in 30 min, λ = 214 nm) of **9a**, His$_6$-SUMO tag cleavage product **9b**, alkylation product **9** and pure **9**. (D) ESI-MS analysis of **9** with the observed mass 15067.0 Da, calcd 15065.5 Da.

1.8 Expression and purification of SelF(U65C/C43S)-CAM mutant

The vector pET-30a (+)-SelF(U65C/C43S) required for the expression of the desired His$_6$-SUMO-SelF(U65C/C43S). The cleavage site between Gly and Phe is marked in bold. The full amino acid sequence of His$_6$-SUMO-SelF(U65C/C43S) was:

MNWSHPQFEKSSGSSGGHHHHHHGGSGGSGSDSEVNQEAKPEVKPEVKPETHINLKVSDGS
SEIFFKIKKTTPLRRLMEAFAKRQGKEMDSLRFLYDGIRIQADQAPEDLDMEDNDIIEAHREQ
IG**GF**GAEFSSEACRELGFSSNLLCSSCDLLGQFNLLQLDPDCRGCCQEEAQFETKKLYAGAIL
EVCGCKLGRFPQVQAFVRSDKPKLFRGLQIKYVRGSDPVLKLLDDNGNIAEELSILKWNTDS
VEEFLSEKLERI

Expression and purification of His$_6$-SUMO-SelF(U65C/C43S):

LB (2 L): 10 g/L tryptone, 5 g/L yeast extract, 10 g/L NaCl, pH 7

cell lysis buffer: 5 mM Imidazole, 0.5 M NaCl, 20 mM Tris, pH 8

Ni-NTA binding buffer: 5 mM Imidazole, 0.5 M NaCl, 20 mM Tris, pH 8

Ni-NTA eluting buffer: 250 mM Imidazole, 0.5 M NaCl, 20 mM Tris, pH 8

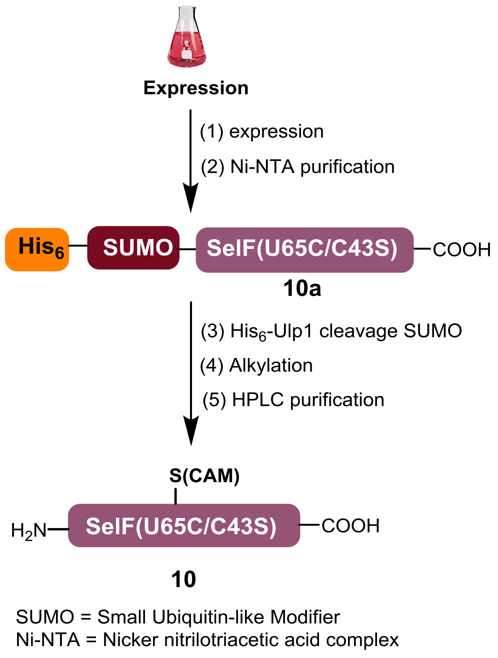

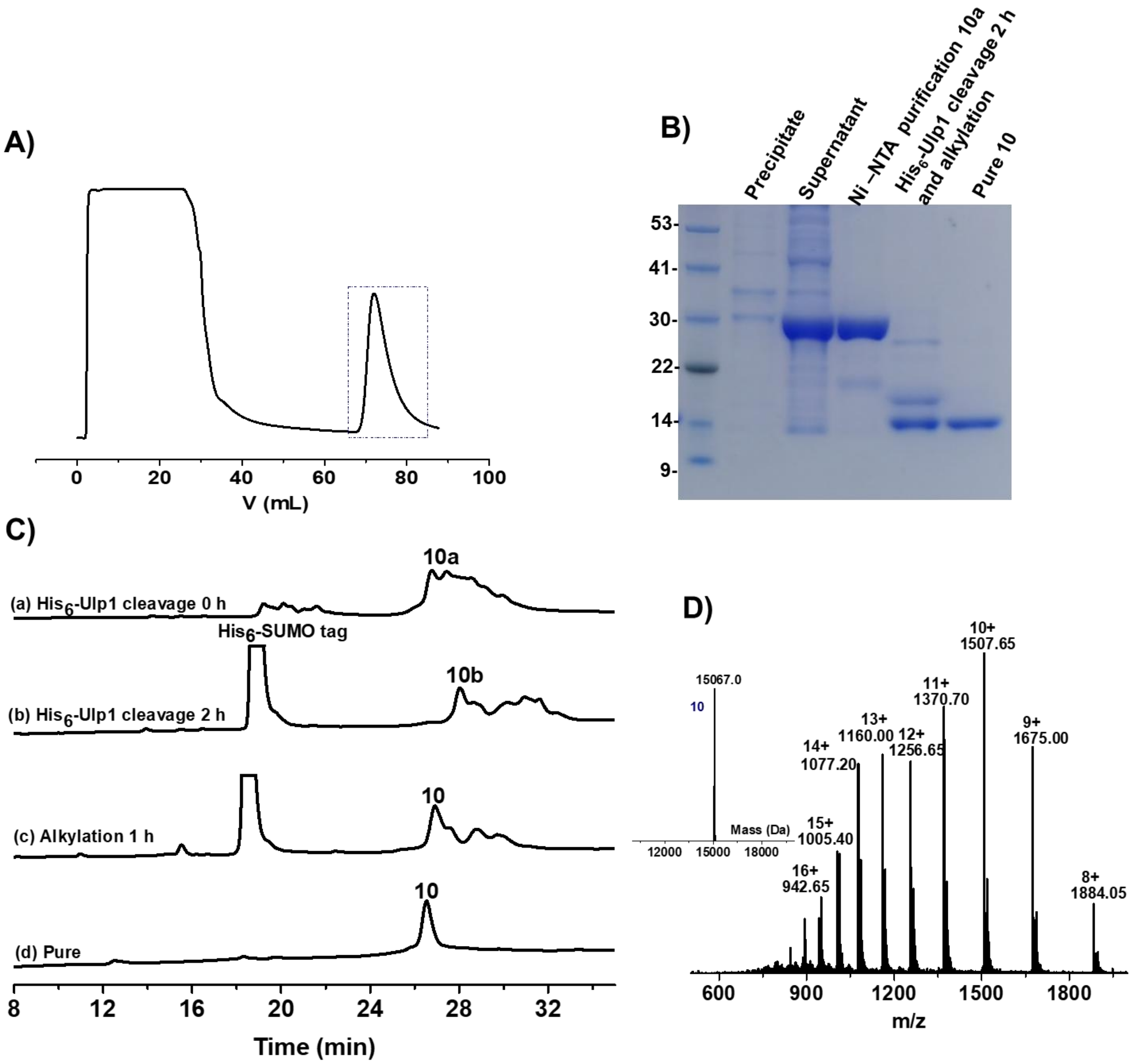

Supplementary Figure 9. (A) Purified by Histrap^{TM} FF column (λ = 280 nm) of His$_6$-SUMO-SelF(U65C/C43S) (**10a**). (B) SDS-PAGE of the His$_6$-SUMO-SelF(U65C/C43S) cell lysis, Ni-NTA purification and **10a** followed by cleavage of the His$_6$-SUMO tag. lane 1: molecular weight standard. Lane 2: precipitate after cell lysis; Lane 3: soluble fraction after cell lysis; lane 4: after Ni-NTA purification of **10a**; lane 5: cleavage His$_6$-SUMO tag 2 h and alkylation 1 h; lane 6: pure SelF(U65C/C43S)-CAM (**10**); (C) Analytic HPLC trace (30 to 55% MeCN (with 0.1% TFA) in 30 min, λ = 214 nm) of **10a**, His$_6$-SUMO tag cleavage product **10b**, alkylation product **10** and pure **10**. (D) ESI-MS analysis of **10** with the observed mass 15067.0, calcd 15065.5 Da.

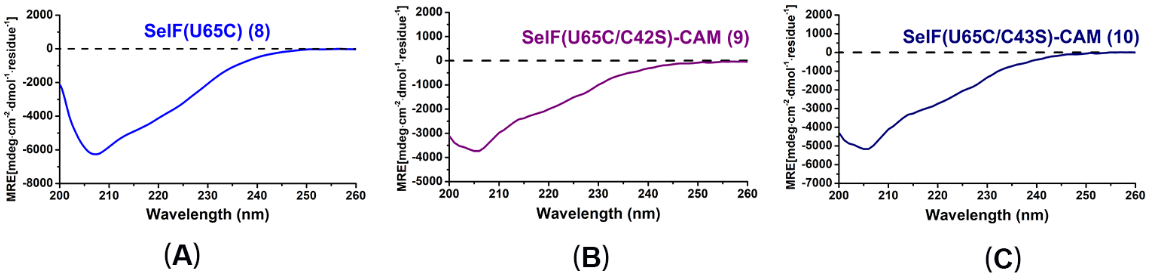

Supplementary Figure 10. CD spectra of the recombinant SelF(U65C) (**8**) (A), the SelF(U65C/C42S)-CAM (**9**) (B) and the SelF(U65C/C43S)-CAM (**10**) (C).

1.9 Enzymatic digestion of SelF(U65C/Q74A)

FGAEFSSEAC^{10}RELGFSSNLLC^{21}SSC^{24}DLLGQFNLLQLDPDC^{39}RGC^{42}C^{43}QEEAQFETKKLYAGAILEVC^{63}GC^{65}
KLGRFPQVA^{74}A^{75}FVRSDKPKLFRGLQIKYVRGSDPVLKLLDDNGNIAEELSILKWNTDSVEEFLSEKLERI^{134}

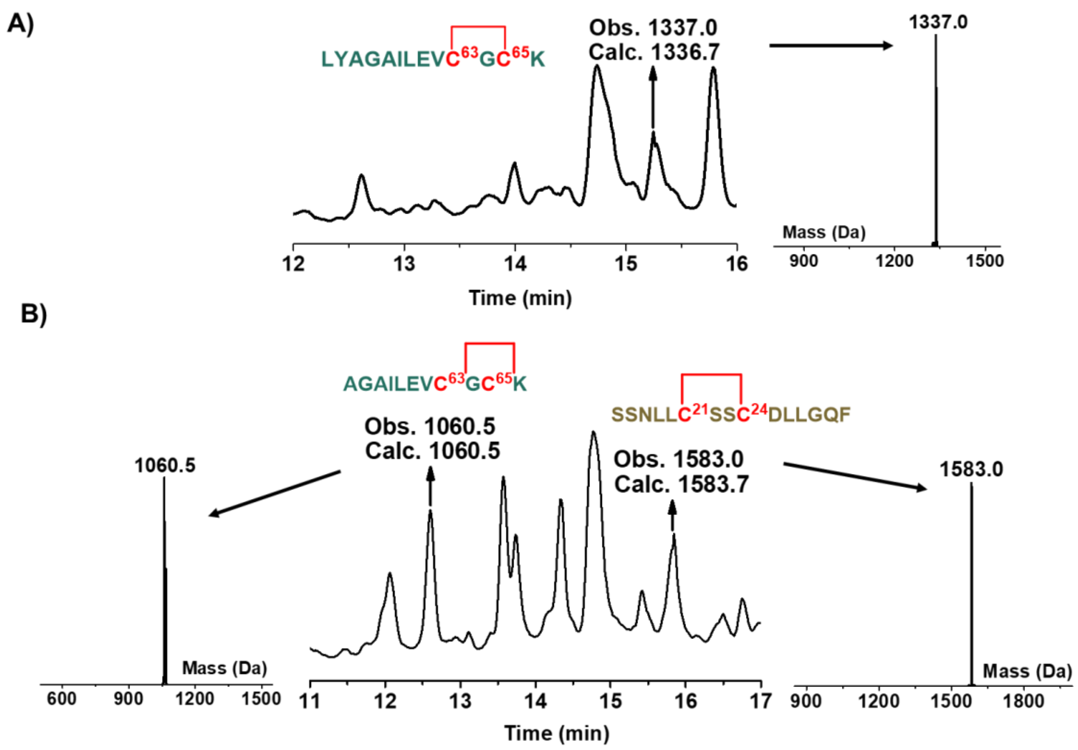

Supplementary Figure 11. (A&B) LC-MS analysis of the trypsin digest (A) and the further digestion with chymotrypsin (B): results obtained with SelF(U65C/Q74A) **(7)**.

1.10 Enzymatic digestion of SelF(U65C)

FGAEFSSEAC^{10}RELGFSSNLLC^{21}SSC^{24}DLLGQFNLLQLDPDC^{39}RGC^{42}C^{43}QEEAQFETKKLYAGAILEVC^{63}GC^{65}
KLGRFPQVQ^{74}A^{75}FVRSDKPKLFRGLQIKYVRGSDPVLKLLDDNGNIAEELSILKWNTDSVEEFLSEKLERI^{134}

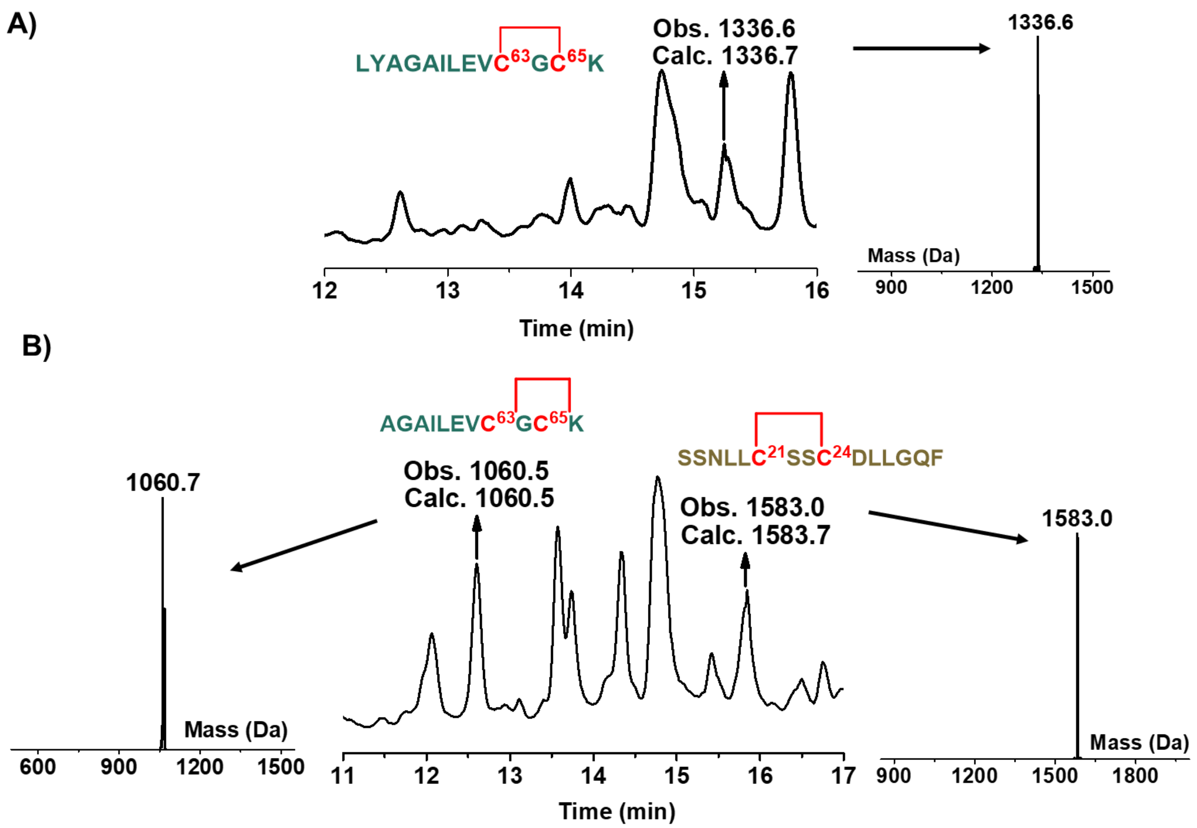

Supplementary Figure 12. (A&B) LC-MS analysis of the trypsin digest (A) and the further digestion with chymotrypsin (B): results obtained with SelF(U65C) (**8**).

1.11 Enzymatic digestion of SelF(U65C/C42S)-CAM

FGAEFSSEAC^{10}RELGFSSNLLC^{21}SSC^{24}DLLGQFNLLQLDPDC^{39}RGS^{42}C^{43}QEEAQFETKKLYAGAILEVC^{63}GC^{65}
KLGRFPQVQ^{74}A^{75}FVRSDKPKLFRGLQIKYVRGSDPVLKLLDDNGNIAEELSILKWNTDSVEEFLSEKLERI^{134}

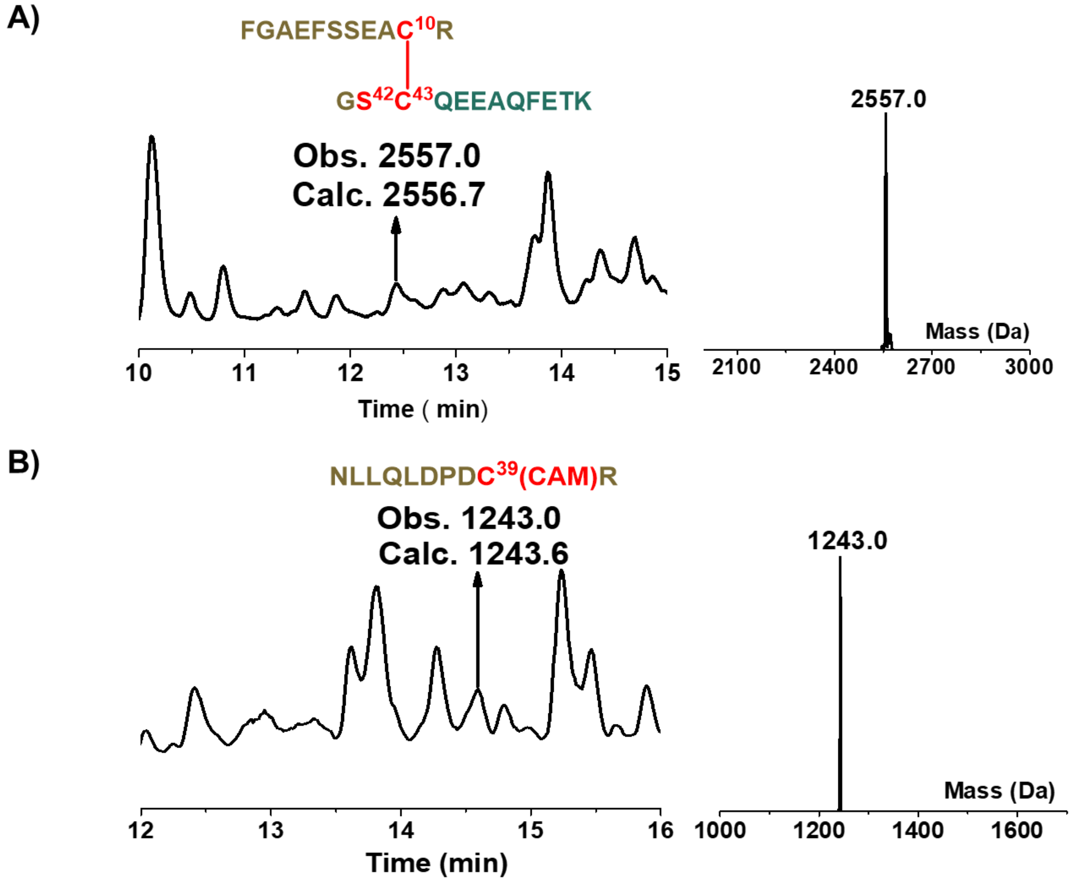

Supplementary Figure 13. (A&B) LC-MS analysis of the trypsin digest (A) and the further digestion with chymotrypsin (B): results obtained with SelF(U65C/C42S)-CAM (**9**).

1.12 Enzymatic digestion of SelF(U65C/C43S)-CAM

FGAEFSSEAC^{10}RELGFSSNLLC^{21}SSC^{24}DLLGQFNLLQLDPDC^{39}RGC^{42}S^{43}QEEAQFETKKLYAGAILEVC^{63}GC^{65}
KLGRFPQVQ^{74}A^{75}FVRSDKPKLFRGLQIKYVRGSDPVLKLLDDNGNIAEELSILKWNTDSVEEFLSEKLERI^{134}

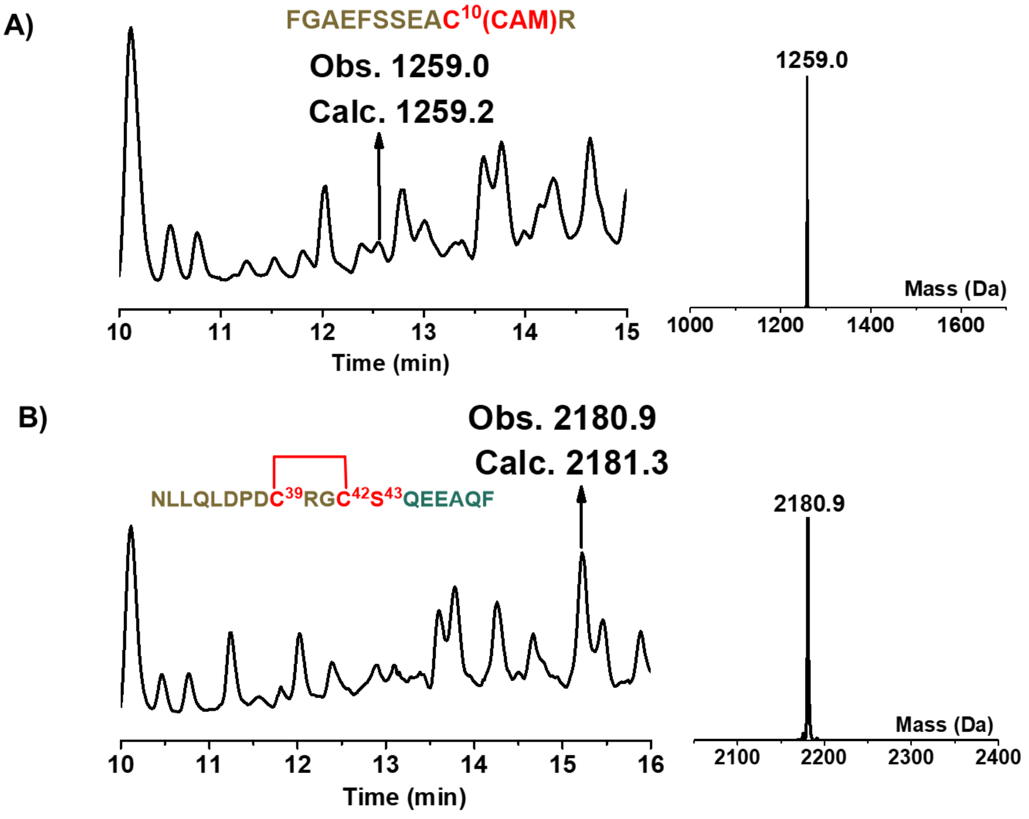

Supplementary Figure 14. (A&B) LC-MS analysis of the trypsin digest (A) and the further digestion with chymotrypsin (B): results obtained with SelF(U65C/C43S)-CAM (**10**).
